# How do Antimicrobial Peptides Interact with the Outer Membrane of Gram-Negative Bacteria? Role of Lipopolysaccharides in Peptide Binding, Anchoring, and Penetration

**DOI:** 10.1021/acsinfecdis.3c00673

**Published:** 2024-01-23

**Authors:** Justus C. Stephani, Luca Gerhards, Bishoy Khairalla, Ilia A. Solov’yov, Izabella Brand

**Affiliations:** † Institute of Physics, Carl von Ossietzky University of Oldenburg, 26111 Oldenburg, Germany; ‡ Department of Chemistry, Carl von Ossietzky University of Oldenburg, 26111 Oldenburg, Germany; § Research Center Neurosensory Science, Carl von Ossietzky University of Oldenburg, 26111 Oldenburg, Germany; ∥ CeNaD—Center for Nanoscale Dynamics, Carl von Ossietzky University of Oldenburg, 26111 Oldenburg, Germany

**Keywords:** Gram-negative bacteria, outer membrane, antimicrobial peptides, lipid–peptide interaction, infrared spectroscopy, spectroelectrochemistry, molecular modeling

## Abstract

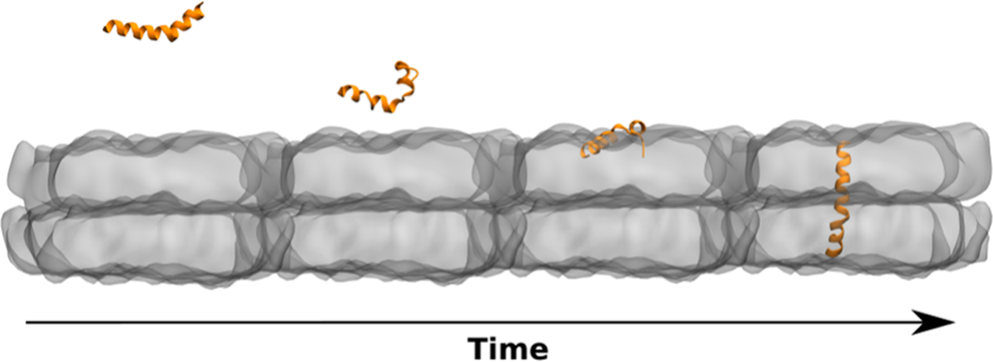

Gram-negative bacteria possess a complex structural cell envelope that constitutes a barrier for antimicrobial peptides that neutralize the microbes by disrupting their cell membranes. Computational and experimental approaches were used to study a model outer membrane interaction with an antimicrobial peptide, melittin. The investigated membrane included di[3-deoxy-d-manno-octulosonyl]-lipid A (KLA) in the outer leaflet and 1-palmitoyl-2-oleoyl-*sn*-glycero-3-phosphoethanolamine (POPE) in the inner leaflet. Molecular dynamics simulations revealed that the positively charged helical C-terminus of melittin anchors rapidly into the hydrophilic headgroup region of KLA, while the flexible N-terminus makes contacts with the phosphate groups of KLA, supporting melittin penetration into the boundary between the hydrophilic and hydrophobic regions of the lipids. Electrochemical techniques confirmed the binding of melittin to the model membrane. To probe the peptide conformation and orientation during interaction with the membrane, polarization modulation infrared reflection absorption spectroscopy was used. The measurements revealed conformational changes in the peptide, accompanied by reorientation and translocation of the peptide at the membrane surface. The study suggests that melittin insertion into the outer membrane affects its permeability and capacitance but does not disturb the membrane’s bilayer structure, indicating a distinct mechanism of the peptide action on the outer membrane of Gram-negative bacteria.

The increasing prevalence of Gram-negative bacteria to antibiotics imposes urgency on research for alternative ways of fighting bacterial infections.^1−4^ Antimicrobial peptides (AMPs) belong to the innate immune system of animals and plants and are promising candidates for new antibiotic therapeutics. These peptides have a broad spectrum of activity against bacteria, viruses, fungi, parasites, and cancer cells. In addition, the development of antimicrobial resistance against AMPs is less probable than that against conventional antibiotics. Therefore, naturally occurring AMPs, their mutants, and synthetic peptides are intensively screened as potential therapeutics.^1,4,5^ AMPs neutralize microbes using different mechanisms of action. Most AMPs interact with the bacterial cell envelope, increasing the membrane permeability that leads to cytoplasm leakage and cell death. However, some AMPs cross the membrane and enter the cell. The intracellular activity of AMPs involves their binding to either DNA or proteins.^6−9^ AMPs are short, positively charged peptides that are characterized by large conformational flexibility and usually have an undefined secondary structure in water. Electrostatic interactions constitute the driving force for anchoring the AMPs on the membrane surface.^4,7,8^ Membrane-associated AMPs interact with the lipid molecules, inserting themselves into the hydrophobic membrane fragment while undergoing a conformational transformation stabilizing α-helical, β-sheet, or extended secondary structure motifs.^10−14^ Inserted AMPs disrupt the phospholipid membrane through the formation of pores, channels, or lipid–peptide micelles.^14−17^ Due to experimental difficulties in the fabrication of models of microbial cell membranes, most of the earlier studies on the action of AMPs were done on phospholipid bilayers, which only remotely resemble models of bacterial cell envelopes.

Melittin is the main component of the venom of western honey bees (*Apis mellifera*).^18^ This 26-amino-acid long AMP is an amphiphilic molecule with a hydrophobic N-terminus and a cationic, polar C-terminus. The peptide includes a proline residue at position 14, which is responsible for a bend between two helical fragments.^18,19^ The presence of proline in the peptide sequence seems to enhance the antimicrobial activity of AMPs.^2^ Melittin became a model AMP for the design of synthetic peptides having a broad cytolytic spectrum,^2,3,5^ and is also a common peptide for biophysical studies of AMP interaction with models of biological cell membranes.^20−26^ In aqueous solutions, melittin monomers adopt a random coil conformation, while in solutions of high ionic strength or peptide concentration, melittin aggregates into α-helical tetramers.^19^ Binding of melittin to a lipid bilayer is coupled with its folding into an α-helix.

The mechanism of the membrane disruption by melittin depends on two main factors: the peptide concentration and the lipid composition of the membrane.^15,20,27,28^ In the initial stages of the interaction, melittin accumulates on the membrane surface. After reaching a certain coverage on the membrane surface, the hydrophobic N-terminus of melittin inserts into the hydrocarbon chain region of a lipid bilayer, forming defects and pores.^15,23,24^ Several mechanisms of the phospholipid membrane lysis by melittin have been described,^3,10,15^ however, the molecular and mechanistic knowledge on cytotoxic activity of melittin on Gram-negative bacteria is still not understood.

Gram-negative bacteria possess two membranes, the outer membrane (OM) and the inner membrane, which are separated from each other by ca. 7 nm thick peptidoglycan layer.^29,30^ The OM is asymmetric and has an exceptional lipid composition, while the inner leaflet contains phospholipids. The OM leaflet is built of lipopolysaccharides, where Lipid A forms the amphiphilic part of each lipopolysaccharide. The polar headgroup of lipopolysaccharides is composed of saccharides that are subdivided into an inner core, an outer core, and eventually O-antigen fragments. The carboxylate, phosphate, and hydroxyl groups in the polar part of the lipopolysaccharides bind electrostatically divalent cations (Mg^2+^, Ca^2+^) to yield a rigid outer leaflet of the OM.^31−33^ Due to the structural and compositional complexity of the bacterial cell envelope, the experimental characterization of its structure, physiochemical properties, and functions are challenging.^33−35^ Computational approaches provide an emerging alternative to gain further insights into the structure, permeability, lipid mobility, and hydration of lipopolysaccharides in the OM.^36−43^

Molecular dynamics simulations and experimental results indicate that the action of AMPs on the OM is different as compared to the phospholipid bilayers.^7,16,38,42−47^ Sharma et al. demonstrated that for a random coil, CM15 peptide approaching the external polar saccharide O-antigen and core regions of lipopolysaccharides preserves the peptide structure characteristic for aqueous solutions.^42^ The peptide penetrates the interfacial region of the inner core, forming hydrogen bonds to phosphate groups of lipid A. It was furthermore revealed that the CM15 peptide adopts a helical conformation upon entering the hydrophobic part of the membrane, which was also observed in another study for the LL-37 peptide interacting with the OM.^45^ The computational study of the CM15 peptide suggested that it may pass through the OM without disrupting it;^42^ it was hypothesized that the CM15 peptide, by lysing the inner phospholipid membrane, kills a microbe. Agglutination was proposed as another possible pathway of AMPs’ action on the bacterial membranes.^9,46,48^

Recent studies show that amyloid peptides^9^ and β-hairpin AMPs,^48^ capable of self-assembly into fibrils, are potent antimicrobial agents. A strong electrostatic binding of AMPs to lipopolysaccharides reduces the peptide mobility, leading to its accumulation in the OM region, which, by agglutination, eventually induces bacterial cell death.^9,48^ The lack of clarity on the action of AMPs on lipopolysaccharides in the OM calls for an in-depth analysis of the underlying molecular interactions.

In the present investigation, we have studied melittin interaction with a model OM consisting of di[3-deoxy-d-manno-octulosonyl]-lipid A (KLA) in the outer leaflet and 1-palmitoyl-2-oleoyl-*sn*-glycero-3-phosphoethanolamine (POPE) in the inner leaflet as illustrated in Figure 1. Due to the large size and structural complexity of the polar part of lipopolysaccharides, biomimetic models are unstable and suffer from a flip-flop of the lipopolysaccharides to the inner phospholipid leaflet.^34^ The KLA–POPE bilayer used in this study is stable and fully asymmetric and includes the lipid A with saccharide residues, representing characteristic features of the OM. Molecular dynamics (MD) simulations were employed to describe the OM interaction with melittin at the atomic level and to characterize the initial phases of melittin binding to the KLA–POPE model OM. The electrostatic interactions anchored the polar, positively charged C-terminus in the polar region of the KLA leaflet, while the N-terminus displayed conformational and motional flexibility. The characteristic peptide conformation and orientation with respect to the OM surface have been determined. Long-time effects of the melittin binding to the KLA–POPE model OM were studied experimentally using in situ polarization modulation infrared reflection absorption spectroscopy (PM IRRAS) with electrochemical control. The results reveal that the electrostatic interactions between melittin and KLA affect the membrane stability and permeability. The association of melittin with the polar parts of KLA involves a partial refolding of the peptide. Over time, melittin adopts an α-helical conformation and moves from the polar head groups in KLA to the hydrophobic core of the membrane, changing the helix orientation from a tilted to normal to the membrane surface.

**Figure 1:**
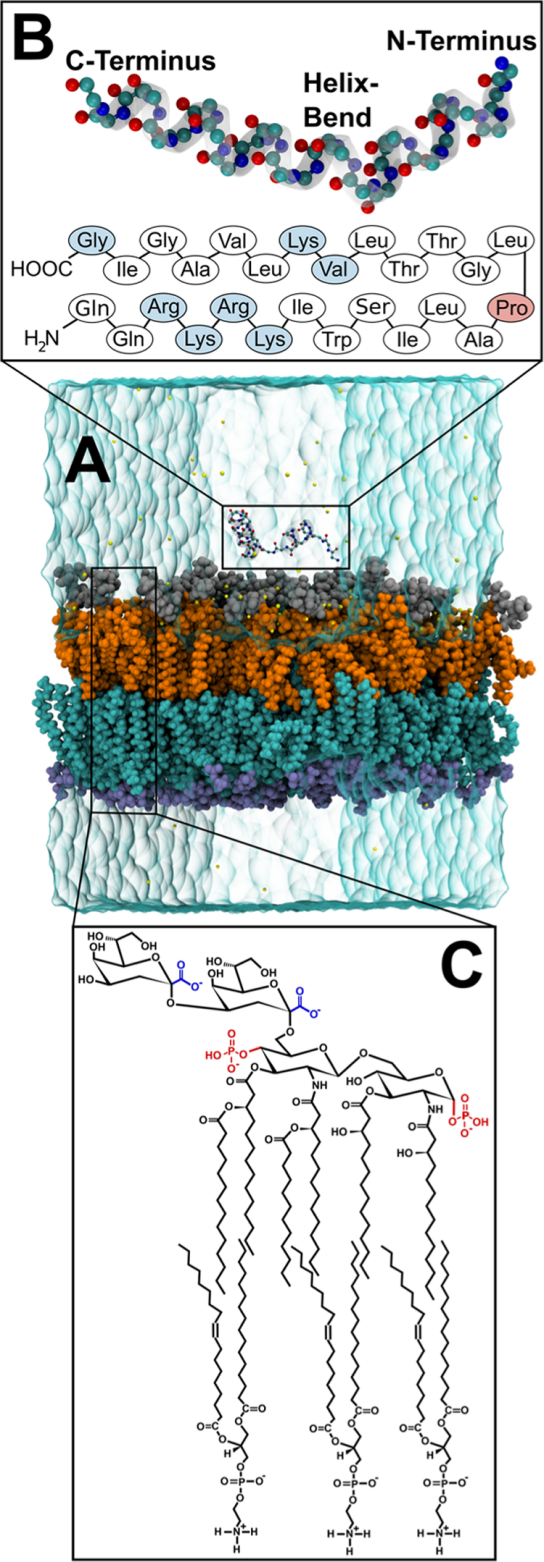
(A) Molecular rendering of melittin in a solvated membrane system. The carbon, oxygen, and nitrogen atoms of the peptide are shown in cyan, red, and blue, respectively. The different layers of the membrane, from bottom to top, are the hydrophilic heads (purple) and the acyl chains (cyan) in POPE phospholipids, the lipid A (orange), and the inner core saccharides (silver) in KLA. Ions of various types are displayed as yellow spheres. (B) Enlarged view of peptide melittin and its primary sequence. The amino acids with the maximum contribution to the interaction energy are shown in light blue, Proline (Pro), where the helix bend in melittin occurs, is highlighted in red. (C) The chemical structure of the membrane lipids KLA (top) and POPE (bottom). The phosphate groups and carboxylate groups in KLA are highlighted in red and blue, respectively.

## Results and Discussion

### Electrochemical Characterization of the Model Outer Membrane Interacting with Melittin

Electrochemical measurements provide information about the compactness and stability of model lipid membranes under changing electric fields. The capacitance of the Au electrode depends on the chemical nature of the adsorbed species, their surface coverage, and packing. The capacitance changes as a function of the applied potential. The difference between the applied potential (*E*) and the potential of zero charge (*E*_pzc_) is an adequate approximation of the membrane potential (*E*^m^),^49,50^ which reads as
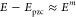
1

**Figure 2:**
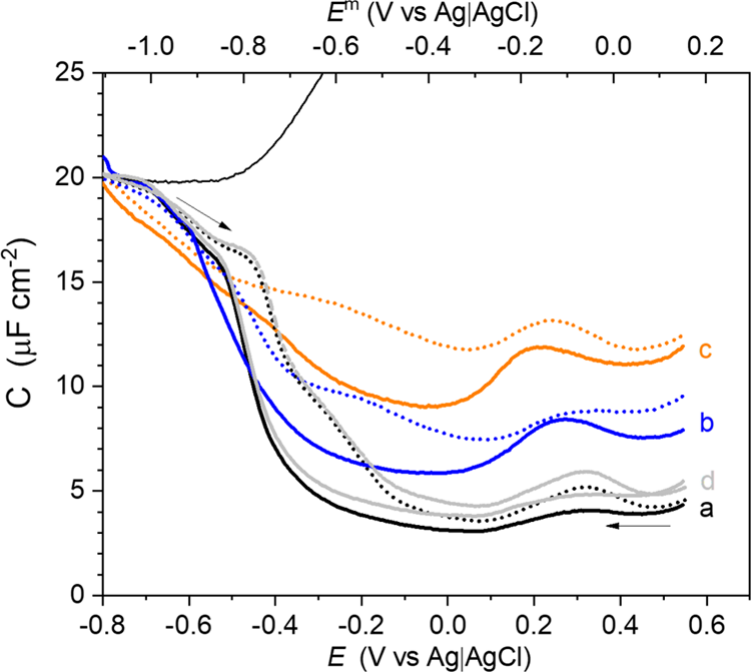
Capacitance (*C*) versus potential (*E*) and membrane potential (*E*^m^) of the KLA–POPE (a–c) bilayers on the Au(111) electrode surface. The measurements were done in the absence of melittin (a, black) after 15 min of interaction with 1 μM melittin (b, blue) and 15 min of interaction with 10 μM melittin (c, orange). Capacitance for the specially prepared LB–LS KLA:Mel (9:1 mol ratio)-POPE bilayer is shown (d, gray). Solid and dotted lines correspond to the negative and positive-going potential scans, respectively. Arrows show the directions of the potential scans. Thin black line: Capacitance of the unmodified Au(111) electrode. 50 mM KClO_4_ and 5 mM Mg(ClO_4_)_2_ was used as the electrolyte solution.

The membrane potential has an important biological significance and indicates direct changes in cell membranes.^51^Figure 2 shows the capacitance of the KLA–POPE bilayers with and without the bound melittin. The capacitance of a lipid bilayer depends on the applied electric potential (membrane potential), which in turn determines the orientation and magnitude of the electric field acting on lipids and proteins in a membrane. Biological membranes, due to the presence of aligned charged lipids and proteins, are typically exposed to high electric fields. Assuming an average thickness of a biological cell membrane of 6 nm and a potential drop of 0.09 to 0.2 V, the electric fields acting at natural cell membranes are in the order of 1–3 × 10^7^ V m^–1^.^51^ Note that the membrane potential may increase to ca. 1 V, resulting in electric fields of 1.5 × 10^8^ V m^–1^. These electric fields may affect the orientation of surface dipoles and lead to charge separation or changes in the hydrogen bond network at the interface, affecting the membrane capacitance. Exposure of the OM to melittin changed the electrochemical properties of the membrane in a peptide concentration-dependent manner. In one set of experiments, the OM was immersed for 15 min into a solution containing either 1 or 10 μM melittin. Afterward, these membranes were transferred to the electrolyte solution containing 50 mM KClO_4_ and 5 mM Mg(ClO_4_)_2_. An increase in the melittin concentration in the electrolyte solution leads to an increase in the membrane capacitance (Figure 2, lines a–c). A broad capacitance maximum appears at −0.30 V < *E*^m^ < 0.05 V, indicating a phase transition and/or a molecular-scale rearrangement in the bilayer. In the OM with melittin associated with the solution phase, the membrane potential of this peak shifts toward more negative values compared to the pure KLA–POPE bilayer (Figure 2, line a). At *E*^m^ < −0.8 V, the measured capacitance was negligibly affected by the presence of melittin (Figure 2, lines a–c). This result means that the presence of melittin has no effect on the potential-driven desorption and disruption of the OM. The interaction of melittin with the OM affected the membrane capacitance, indicating changes in its permeability and stability; however, it did not lead to the dissolution of the lipid bilayer from the Au(111) surface.

In the second set of experiments, a KLA/melittin (9:1 mol ratio) monolayer was transferred by the LS method onto the Au(111) surface covered by the inner POPE leaflet. In this case, the electrochemical characterization of the lipid–peptide membrane was comparable to that of the pure KLA–POPE bilayer (Figure 2, lines a and d), indicating an insignificant effect of melittin on the electrochemical properties of the OM. Thus, the interaction pathway of melittin with the OM depends on the delivery strategy of the peptide.

Melittin may either accumulate on the surface of the KLA leaflet or penetrate into the OM, forming defects and channels as schematically shown in Figure S3. Electrochemical measurements are sensitive to different supramolecular arrangements of molecules in a film covering an electrode surface. The dielectric constant of the adsorbing molecule has an impact on the capacitance value; see Section S3. Lipid molecules are amphiphilic and contain long acyl chains. The dielectric constant of a hydrocarbon chain is ∼2 while that of the polar headgroup of lipids is 5–8;^52^ the dielectric constant of proteins is difficult to estimate and was reported to be in the range 7–30.^53^ Experimentally measured capacitance of the OM-melittin films contains a contribution from the capacitance of the lipid membrane (*C*_m_), adsorbed peptide (*C*_p_), and the diffuse layer (*C*_dl_), and reads as
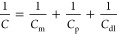
2

Adsorption of melittin on top of the lipid bilayer surface, see Figure S3A, does not affect the measured capacitance because *C* is dominated by the capacitance of the subsystem with the lowest value (*C*_m_). In comparison to a pure KLA–POPE bilayer (see Figure 2, line a), the OM with KLA and melittin transferred by the LB method (see Figure 2, line d) displays minor changes in the capacitance. The spectroscopic studies revealed that a freshly transferred KLA/melittin (9:1 mol ratio)-POPE bilayer contains melittin; see Figure S4, gray line. However, after immersion of the bilayer into the electrolyte solution, the intensity of the amide I’ mode of melittin decreased significantly; see Figure S4, black line. This result indicates that melittin is weakly associated with the bilayer; it accumulates in the polar headgroup region (on top) of the KLA leaflet and does not enter the hydrophobic fragment of the KLA lipid. In contrast, an increase in the capacitance observed upon melittin binding from the solution, compared to the pure KLA–POPE bilayer, indicated an insertion of the peptide into the membrane, see Figure S3B,C. The membrane insertion of melittin is expected to affect the packing and orientation of the lipid molecules and change the conformation and/or hydration of the peptide in the environment of the OM. The electrochemical data suggest molecular-scale rearrangements in the orientation and conformation of lipids and melittin during interaction. To describe this lipid–peptide interaction at molecular and atomic levels, MD simulation (short interaction time) and in situ spectroelectrochemical (long interaction time) experiments were performed.

### Melittin Binding, Conformation, and Orientation: A Short-Time Scale of Interaction

**Figure 3:**
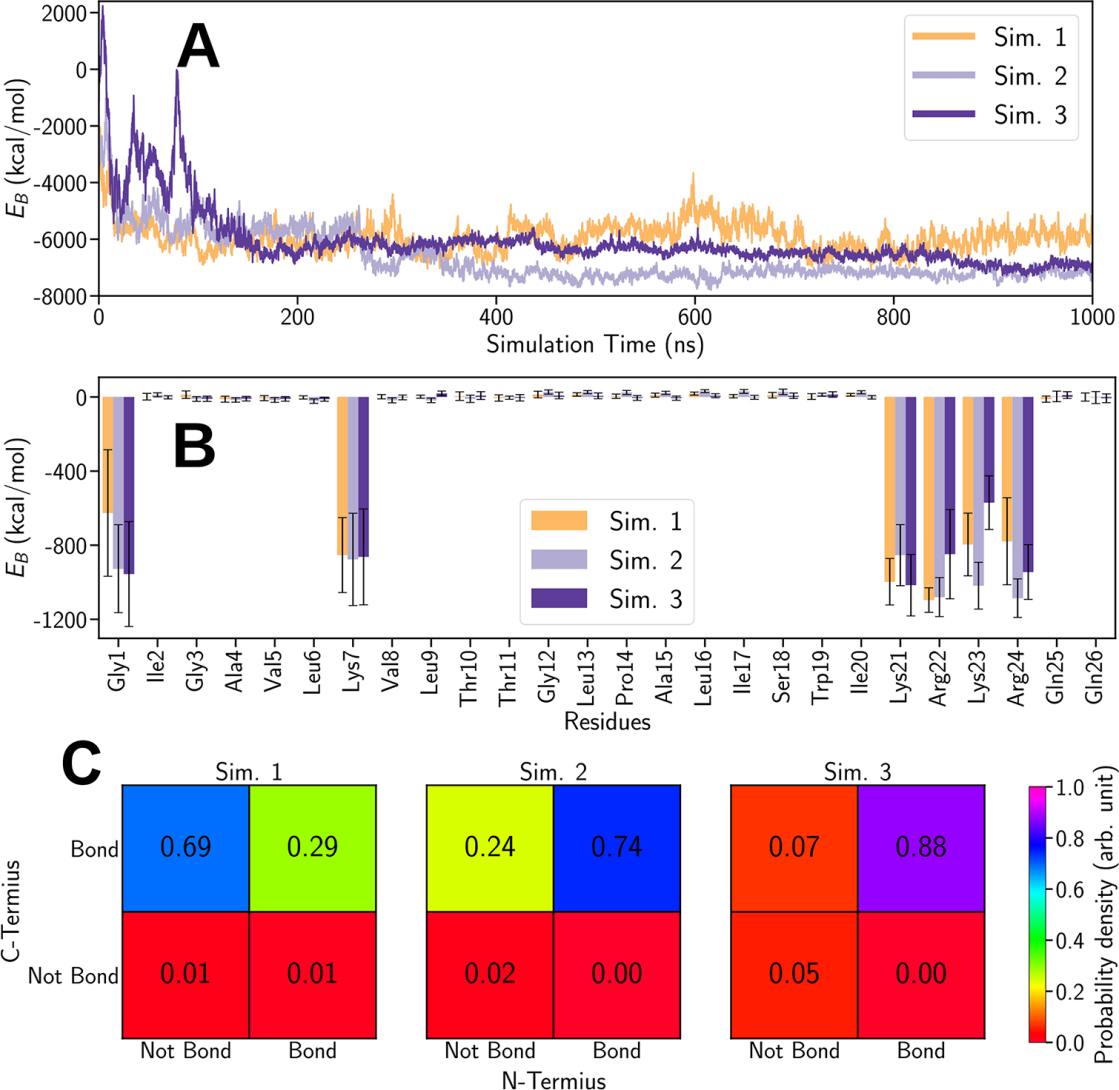
Interaction energy *E*_B_ between melittin and the KLA–POPE membrane calculated for the simulations Sim. 1, Sim. 2, and Sim. 3. (A) Temporal evolution of *E*_B_. (B) Time-averaged values of *E*_B_ were computed for each individual amino acid residue in the peptide. The standard deviations are shown as error bars. (C) Binary probabilities of the C-/N-terminus of melittin binding to the membrane in the three simulations.

Three independent simulations (Sim. 1–3, see Figure 9 in the Methods Section) show melittin binding to the model membrane. To gain insight into the binding mechanism, the interaction energies between the individual amino acids in the peptide and the membrane were calculated. Interaction energies include Coulomb and dispersive (van der Waals and hydrophobic) contributions. The details of the calculation of the contributions to the interaction energy are described in the Supporting Information S5. Figure 3A shows the time evolution of the total interaction energy (*E*_B_) between the peptide and the membrane for all three simulations (Sim. 1–3). The figure features a decrease in the interaction energy in all simulations, which indicates the binding of melittin to the KLA leaflet. Note that the different initial orientations of the peptide with respect to the membrane did not significantly affect the resulting melittin binding energy; however, it affected how fast the stable value was reached. Comparing the results of Sim. 3 (purple) with Sim. 1 (orange) and Sim. 2 (lilac), it is clear that the peptide in Sim. 3 experienced an unfavorable initial orientation of the N-terminus above the membrane surface. In this case, during the first 100 ns of the simulation, the peptide moved away from the membrane, reoriented, and finally bound with its C-terminus to the membrane. It is worth noting that in Sim. 1, the N-terminus detaches from the membrane while the peptide still stays bound to the surface through its C-terminus; such behavior is not present in Sim. 2 and 3. Figure 3A,B supports this observation as the interaction energy of melittin with the membrane slightly increases over time in Sim. 1 and the partial interaction energy of the Gly1 residue is significantly lower compared to the other simulations. The performed three independent replicas, 1 μs long simulations, are sufficiently long to explore the possible conformational dynamics of a 26-amino-acid residue long peptide; this time scale is comparable with the characteristic folding time of small proteins.^54,55^ Therefore, although this time scale is orders of magnitude lower than in experiments, it still permits one to judge about melittin binding modes to the membrane surface on an atomistic scale and to explore the details of the relevant potential energy landscape. The differences in the three simulations clearly indicate an intrinsic flexibility of the membrane-attached peptide.

Figure 3C shows the probabilities of C-/N-terminus binding to the membrane in the three simulations. The plots illustrate the four possible scenarios of the termini being bound, unbound, or partially bound to the membrane. The results indicate that the C-terminus is virtually always bound, while the N-terminus is notably bound in Sim. 2 and 3 and weakly bound in Sim. 1.

The individual contributions of each residue to the overall interaction energy are shown in Figure 3B. From the 26 residues of melittin, only six contributed significantly to the overall interaction energy: Gly1, Lys7, Lys21, Arg22, Lys23, and Arg24. The positively charged Lys and Arg residues (21–24) present in the helix of the C-terminus bind the peptide to the OM. Hydrogen bonds between the residues Lys21, Arg22, Lys23, and Arg24 of the peptide and the KLA in the OM were observed. The ε-ammonium group in Lys and the guanidinium group in Arg acted as the hydrogen donors, while the oxygen atoms of carboxylate, phosphate, and hydroxy groups in KLA accepted the hydrogen atoms. The measured donor–acceptor distances were between 2.2 and 3.0 Å, depending on the specific groups that formed the hydrogen bond. With advancing simulation time, these bonds were observed more frequently, indicating an increase in the stability of the bonds and thereby explaining the strong binding of the helical structure at the C-terminus of melittin to the OM. At the N-terminus, only Gly1 and Lys7 residues interacted with the membrane. In the control simulation performed in water, a loss of helicity from initial 75% to ca. 35% over 1 μs was observed, see Figure S5A. The binding of melittin to the OM is connected with the stabilization of the α-helix secondary structure. In the three simulations, the content of the α-helix secondary structure varied between 35 and 70%, underlining the conformational flexibility of the peptide.

**Figure 4:**
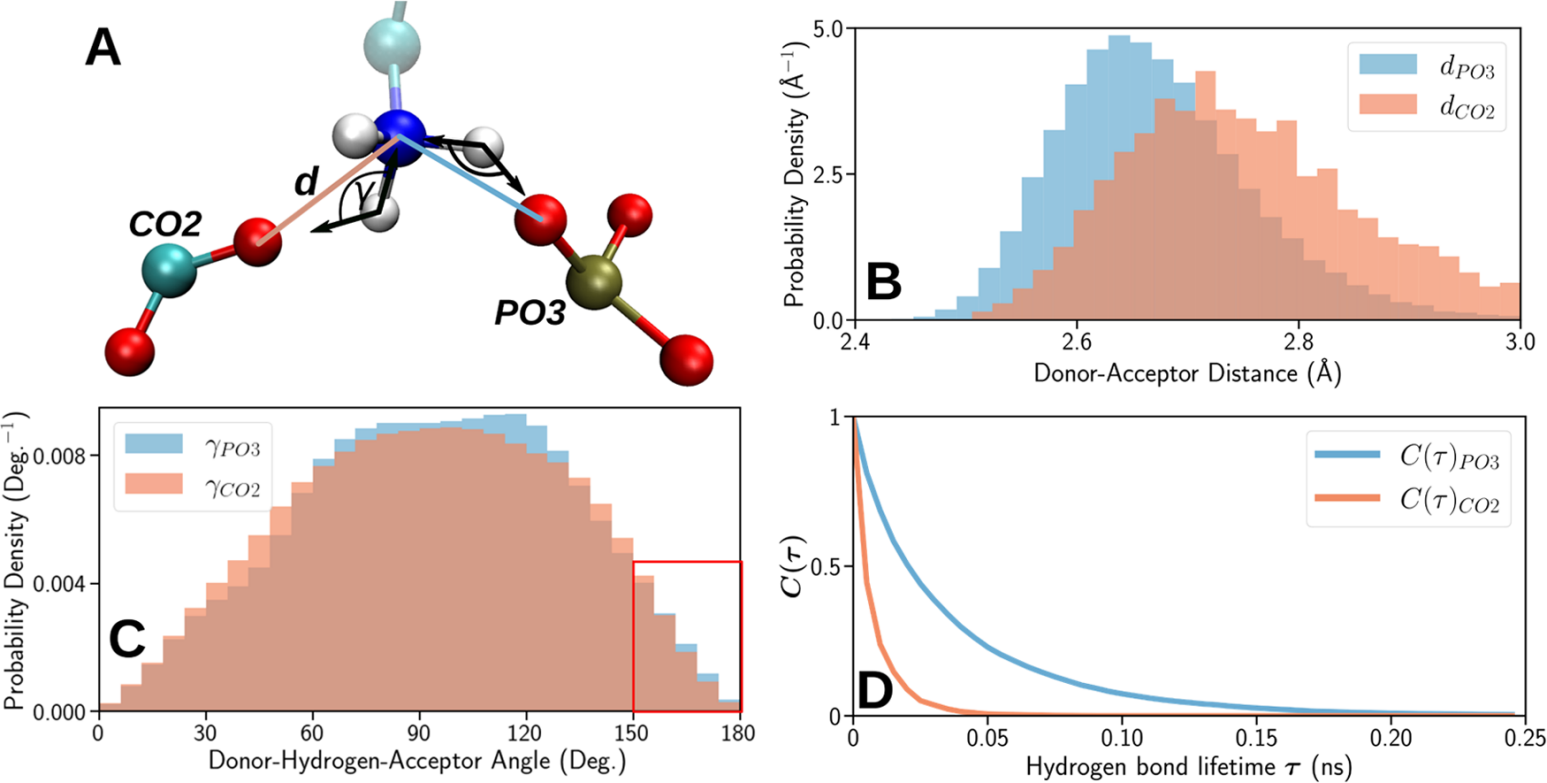
(A) Rendering of a phosphate group in the lipid A part of a carboxylate group in the inner core in KLA in close proximity to the N-terminus of melittin. The nitrogen, carbon, phosphorus, oxygen, and hydrogen atoms are shown in blue, cyan, ocher, red, and white, respectively. The minimal distances to the carboxylate group and to the phosphate group are labeled *d*_PO_3__ and *d*_CO_2__, respectively, and are highlighted in blue and orange. The angle γ between atoms forming the hydrogen bond is indicated. (B) Probability density distributions for the *d*_PO_3__ and *d*_CO_2__ distances corresponding to the formed hydrogen bonds with the involved groups in the case of Sim. 2. (C) Probability density of the angle between the atoms involved in hydrogen bond formation. The angular window relevant to hydrogen bond formation is indicated in red. (D) The hydrogen bond time autocorrelation function, see eq 3, is of the phosphate group *C*_PO_3__(τ) and the carboxylate group *C*_CO_2__(τ).

The interaction of the Gly1 residue in the N-terminus of melittin with the OM was analyzed in detail by studying the formation of hydrogen bonds between the N-terminus and the phosphate group in the lipid A part and carboxylate groups of the inner core in KLA molecules. The results are illustrated in Figure 4. A hydrogen bond is defined by geometric criteria that include the distance between the donor and acceptor atoms and the value of the donor–hydrogen–acceptor planar angle.^56^ The NH_2_ group in Gly1 in the N-terminus and a phosphate or carboxylate group were considered hydrogen-bonded if the donor–acceptor distance *d* was less than 3 Å and the value of the donor–hydrogen–acceptor angle turned out to be 150–180°. Figure 4A shows the typical location of the amide group in Gly1 in the N-terminus of melittin (nitrogen in blue and hydrogen in white) in the KLA leaflet. Gly1 interacts with the phosphate groups of the lipid A part in KLA (phosphorus in ocher and oxygen in red) and the carboxylate groups of the inner core in the KLA molecule (carbon in cyan and oxygen in red). The orange and blue lines in Figure 4A represent the existing donor–acceptor distances between oxygen atoms in the PO_3_ and CO_2_^–^ groups of KLA and the N-terminus of melittin. The orange line represents the minimal donor–acceptor distance to the closest oxygen atom of the CO_2_^–^ group, and the blue line represents the minimal distance to the closest oxygen atom of the PO_3_ group. The probability distribution of distances obtained from Sim. 2, *d*_CO_2__ and *d*_PO_3__, are shown in Figure 4B. The most probable donor–acceptor distance between the NH_3_ group of the Gly1 residue and the oxygen atoms of the phosphate groups is smaller compared to the distance to the carboxylate group. The distance *d*_PO_3__ is rather conserved over a significant simulation time at a value of ∼2.7 Å, while the value of *d*_CO_2__ fluctuates more, indicating a stronger and longer living hydrogen bond to the phosphate groups. The distance between the donor–acceptor sites alone, however, is insufficient to define the lifetime of a hydrogen bond since it also depends on the planar angle formed by the involved groups, see Figure 4C. For further investigations of the hydrogen bond dynamics, the average lifetime (*T*) was calculated through the autocorrelation function defined as^56^
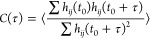
3Here, *h*_*ij*_ (*t*_0_) measures whether a pairing between a hydrogen atom *i* of the N-terminus and the acceptor oxygen atom *j* of the carboxylate or phosphate groups satisfy the hydrogen bond criteria at the reference time instance *t*_0_, while *h*_*ij*_(*t*_0_ + τ) checks for the hydrogen bond’s existence at the time instance *t*_0_ + τ. The summation was carried out over possible pairs *ij* between the hydrogen atoms of the NH_3_ group of Gly1 and the oxygen atoms of either the phosphate group or the carboxylate group of KLA. Angular brackets indicate an average over the different starting time instances.

The computed autocorrelation functions are shown in Figure 4D for the hydrogen bonds formed to the oxygen atoms of the phosphate groups *C*_PO_3__(τ) and to the oxygen atoms of the carboxylate groups *C*_CO_2__(τ). The hydrogen bond lifetime *T* is then defined as the integral of the autocorrelation function^56^ as
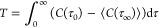
4

The lifetime *T* is calculated by fitting the results of the autocorrelation function with a biexponential function and subsequently numerically integrating eq 4. This yields an average lifetime of 0.035 ns for a hydrogen bond between the N-terminus in melittin and the oxygen atom of a phosphate group in KLA and 0.008 ns for a hydrogen bond with the carboxylate group in KLA. The presence of the charged N-terminus and its binding to the carboxylate and phosphate groups might supersede a divalent cation that binds to a phosphate group or a carboxylate group of the KLA in a melittin-free membrane. The earlier PM IRRAS results indicated that upon melittin binding, the coordination to the carboxylate groups in the inner core of the KLA changes, confirming that the negatively charged residues in KLA interact directly with melittin.^35^ MD simulations also show that the N-terminus in melittin, within 1 μs of the simulation time, interacts with the inner core to make stable hydrogen bonds with the phosphate residues located at the polar–hydrophobic interface of the lipid A part in the KLA, see Figure S6. The binding of melittin to the KLA may change the balance of charges in the outer leaflet of the OM and cause structural disturbances in the lipid membrane, which is an essential part of the insertion of melittin into the membrane.^57^

The tilt angle of the helices in melittin relative to the membrane surface was determined. Each amide group of melittin includes an IR-active C=O bond aligned with the corresponding transition dipole moment. The dipole moment for each residue of melittin is characterized by a tilt angle θ*_n_*, computed relative to the membrane surface normal as
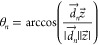
5where *d⃗_n_* is the transition dipole moment of the C=O stretching mode (ν(C=O) mode) in the *n*-th residue and *z⃗* is the normal vector pointing perpendicular to the membrane surface. The resulting time average angle ⟨θ⟩ for the whole melittin can be computed as
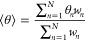
6where the weights *w_n_* describe the coupling of the transition dipole moments of the ν(C=O) modes in melittin to the electric field vector of the reflected IR radiation and are defined as the *z*-component of the normalized vector of the respective dipole moment . The summation was carried out over 3 μs of the combined trajectories of the simulations Sim. 1–3, yielding the average tilt angle of = 44.8°. The tilt of the long axis of the helical fragments in melittin with respect to the membrane surface normal could readily be related to ⟨θ⟩ (see Section S10) and ranges between 31 and 36°.

The time scale of the simulations cannot be directly compared with the time scale of the experiment. This is a very typical situation, as atomistic MD simulations would likely always be orders of magnitude shorter than real live experiments. However, often, useful information could be extracted from the atomistic MD data, which allows for interpretation of certain experiments. This scenario is also exactly the case in our study. In the simulation, we place the peptide directly on the membrane surface. We chose three different initial orientations to probe as many conformational changes (and binding modes) as possible within the limited simulation time. Note that in the simulations, the peptide is already at the membrane surface, while in the experiment, melittin is dissolved in the electrolyte solution and must diffuse to the membrane surface, find the favorable binding orientation, and subsequently undergo conformational and orientational changes. The molecular simulation results show that melittin associates with the membrane surface, anchoring its C-terminus in the polar headgroup region of the KLA leaflet. The more flexible N-terminus may move away from the saccharide groups in KLA, anchoring the peptide at the hydrophilic–hydrophobic interface of the outer leaflet of the outer membrane. Further conformational and orientational changes require longer times, as observed in the experiments.

### Melittin Binding, Conformation, and Orientation: A Long Interaction Time Scale

**Figure 5:**
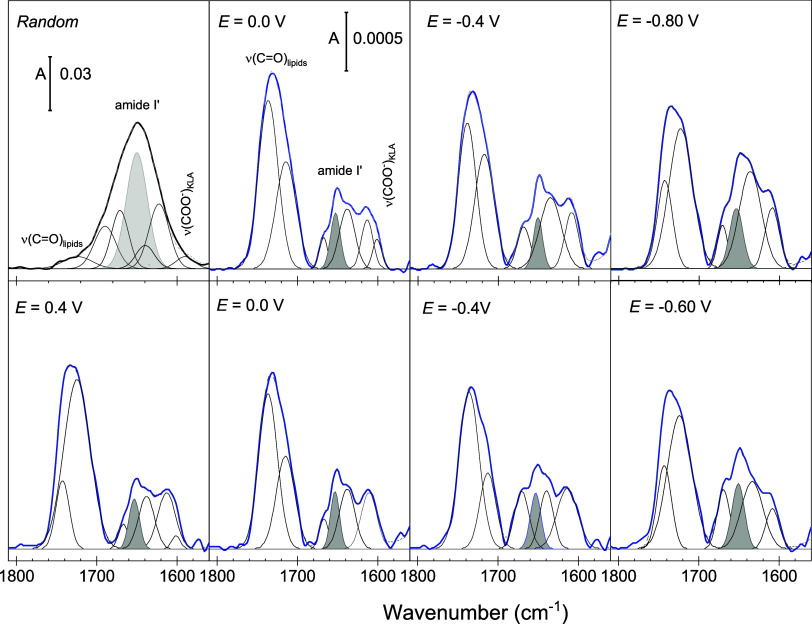
IR spectra of KLA–POPE bilayer systems (thick lines) after the interaction with melittin. The left spectrum in the upper panel shows an ATR IR spectrum of KLA–POPE vesicles after 60 min interaction with 4.4 × 10^–4^ M melittin. Other figures show the PM IRRA spectra of KLA–POPE bilayers after 15 min of interaction with melittin (blue lines) recorded at different electrode potentials. The upper and lower panels show results for the negative and positive scans, respectively. Thin black lines show the band deconvolution results. The bands highlighted in gray show the amide I’ mode of α-helices in melittin. Measurements were carried out for 1 μM melittin in 50 mM KClO_4_ and 5 mM Mg(ClO_4_)_2_ in D_2_O. The absorbance is shown in arbitrary units.

Figure 5 shows the PM IRRA spectra of the KLA–POPE bilayer after 15 min of incubation in 1 μM melittin solution. The spectra feature the ν(C=O) stretching modes in the ester carbonyl groups in lipids, amide I’ vibration mode mainly in melittin, and ν_as_(COO^–^) stretching mode in KLA. Furthermore, two amide groups in KLA also contribute to the amide I’ vibration mode; however, these groups have a slight impact on the resulting IR spectrum.

In the presence of melittin, the ν_as_(COO^–^) absorption bands in KLA appear at 1610–1608 and 1600 cm^–1^. The absorption maximum of the high wavenumber ν_as_(COO^–^) mode is ca. 5 cm^–1^ down-shifted compared to the position of this band in the pure KLA–POPE bilayer,^35,49^ being the result of changes in the coordination to the carboxylate group. The observed spectral changes are associated with the removal of Mg^2+^ ions and the formation of direct hydrogen bonds to positively charged amino acids in melittin.

The amide I’ band in melittin is a complex spectral feature centered at 1650 cm^–1^, see Figure 5. The IR spectrum of melittin KLA–POPE vesicles corresponds to a random distribution of the peptide. The amide I’ band was deconvoluted into five components centered at 1689, 1670, 1650, 1639, and 1622 cm^–1^; see Figures 5 and S7A. The complexity of the amide I’ mode points to structural flexibility and significant differences in the hydration and hydrogen bonding network in melittin in solution and lipid bilayer-associated states, agreeing with earlier findings.^23,24,58−61^ For randomly distributed melittin, the strongest component of the amide I’ band (1650 cm^–1^) stems from α-helices that constitute 42% of the peptide secondary structure. The modes at 1670 and 1630 cm^–1^ could be attributed to either helical (3_10_- or π-) structures or random coils.^60^ Assignment of the IR absorption modes at 1689 and 1622 cm^–1^ is primarily associated with antiparallel β-sheets.^58,61^ Drastic changes in the hydrogen bonding network at the helical and disordered peptide fragments, however, may result in similar spectral changes. Dehydration of the C=O group causes an upshift of the amide I’ mode to 1685–1700 cm^–1^.^62^ A strong hydration of the helical peptide fragments may result in a down-shift of the amide I’ mode to ca. 1625 cm^–1^.^24,60,63^ The IR spectrum of melittin in solution was measured for the peptide:lipid ratio of 1:22. At such a high content of melittin, the peptide may aggregate on the vesicle surface that may induce even a conformational change to β-sheet structures as proposed earlier.^24,58,61^

MD simulations confirm the structural flexibility of nonbonded melittin. In the control simulation, in water, gradual loss of the helical structure to ca. 35% was observed; see Figure S5A. Simultaneously, changes in the backbone dihedral angles indicate that melittin may adopt a β-sheet structure; see Figure S5C. It is therefore reasonable to conclude that melittin approaching the OM surface from the solution phase is characterized by a conformational flexibility and hydrogen bonding network of different strengths.

After 15 min of the OM interaction with 1 μM melittin, the amide I’ band features three components centered at 1669, 1653, and 1636 cm^–1^, see Figures 5 and S7B. The observed spectral changes indicate that membrane-associated melittin is composed of helices and random coils, reflecting only to a certain extent the structural flexibility found in the solvated peptide; see Figure 5. MD simulations confirm the observation, as after 1 μs of interaction, the peptide did not adopt a fully helical conformation. Both experimental and simulation results show that the process of melittin integration into the OM is characterized by a gradual insertion of the peptide into the polar environment of lipid A.

**Figure 6:**
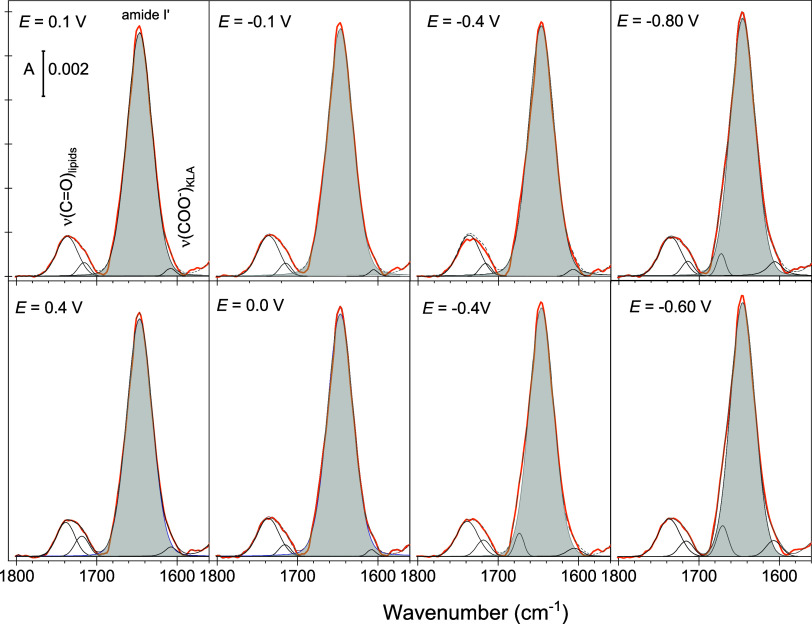
IR spectra of KLA–POPE bilayers on Au(111) after 15 min of interaction with melittin (orange lines) recorded at different electrode potentials. The upper and lower panels show results for the negative and positive scans, respectively. Thin black lines show the band deconvolution results. The bands highlighted in gray show the amide I’ mode of α-helices in melittin. Measurements were carried out for 10 μM melittin in 50 mM KClO_4_ and 5 mM Mg(ClO_4_)_2_ in D_2_O. The absorbance is shown in arbitrary units.

Figure 6 shows the PM IRRA spectra of the OM after 15 min interaction with 10 μM melittin. Independent of the electrode potential, a strong symmetric amide I’ band, centered at 1648 cm^–1^, is present in the spectra. At *E* > −0.4 V, the amide I’ band in melittin contains one component assigned to α-helices. At *E* < −0.4 V, a weak amide I’ band at 1670–1673 cm^–1^ appears in the spectra, indicating the presence of other structural elements in the membrane-bound melittin or the presence of poorly hydrated peptide fragments. Similar behavior was observed for 1 μM melittin interacting with the OM for 60 min (see Figure S8). For both melittin concentrations, the potential-dependent spectral changes were reversible, indicating that melittin reached a steady-state well-defined orientation within the OM. The observed enhancement of the intensity of the amide I’ band of α-helices in melittin (centered at 1648–1654 cm^–1^) and reversibility of the potential-dependent changes in the shape of the entire amide I’ band confirm an anisotropic orientation of the melittin helix with respect to the OM surface, allowing for a quantitative analysis of the helix tilt at different stages of its interaction with the membrane.

**Figure 7:**
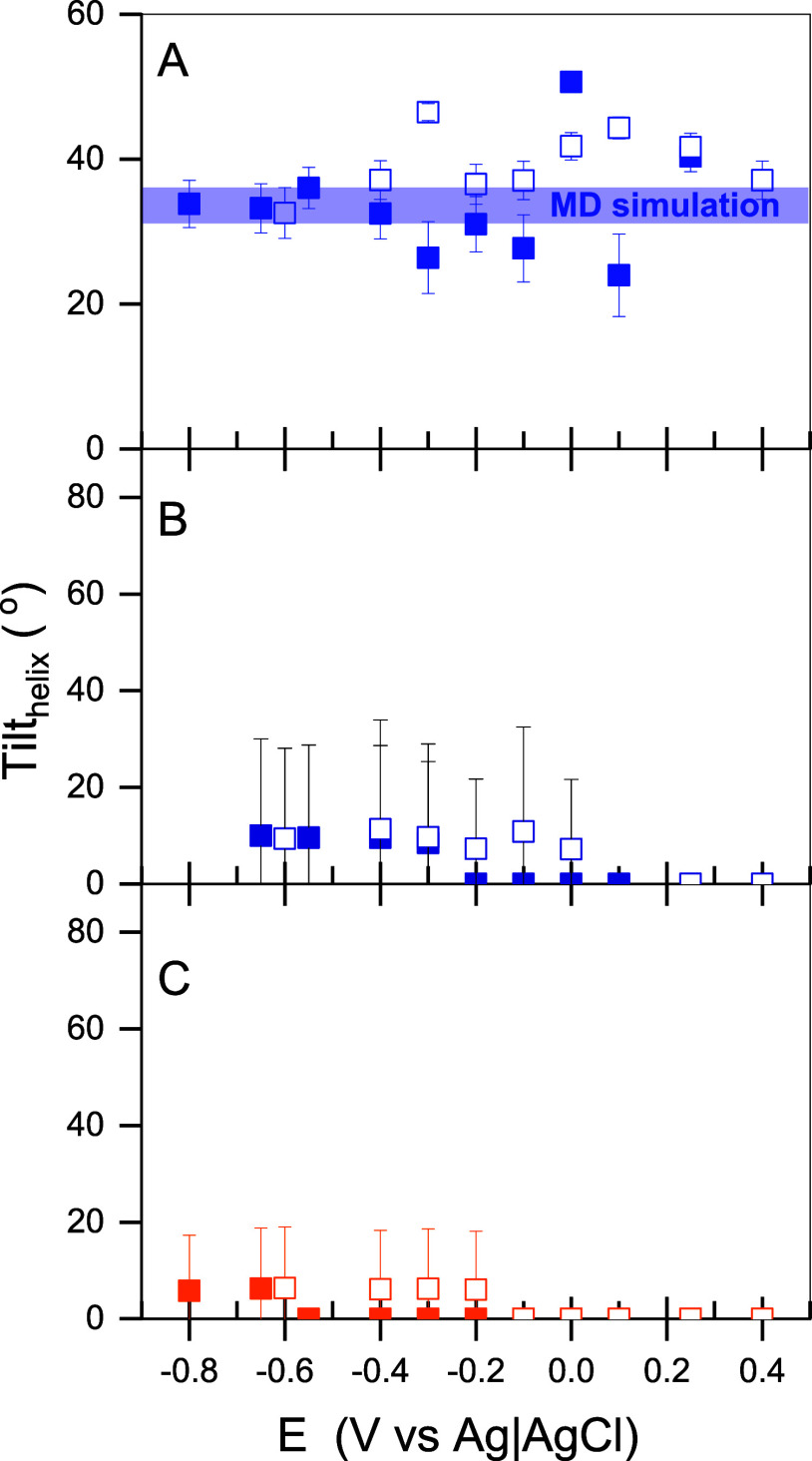
Electrode potential dependence of the tilt angle of the long helix axis in melittin with respect to the KLA–POPE bilayer surface normal. The measurements were done for different melittin concentrations and incubation times as (A) 1 μM for 15 min, (B) 1 μM for 60 min, and (C) 10 μM for 15 min. The electrolyte solution contained 50 mM KClO_4_ and 5 mM Mg(ClO_4_)_2_ in D_2_O. Full and empty symbols indicate the tilt angles determined from negative and positive potential scans, respectively. The blue line in panel A shows the tilt of the helix in melittin obtained from the MD simulations.

The integral intensities of the deconvoluted components of the amide I’ band were used to calculate the average tilt of the long axis of the α-helix in melittin, see Section S10. In this calculation, only the helical fragments of melittin were considered. Different results were obtained for short (15 min) and long (60 min) times of the interaction of 1 μM melittin solution with the model OM, demonstrating the dynamics of folding and reorientation of the peptide associating with a model microbial membrane; see Figure 7. Over the first 15 min of melittin’s interaction with the OM, the average tilt angle of 37 ± 5° for the long axis of the helical peptide fragments relative to the membrane surface normal was determined; see Figure 7A. The MD simulations reveal a similar value of 31–36°, as demonstrated above (see Figure 7A). Thus, the helical melittin fragments, at the beginning of the interaction, adopt a tilted arrangement with respect to the OM surface. Both experimental and simulation results show that the process of melittin integration into the OM is characterized by insertion of the peptide into the polar environment of lipid A. This process is accompanied by changes in the peptide conformation and progressive reorientation of its helical fragments with respect to the membrane normal.

Experimental results show clearly that longer incubation time or higher peptide concentrations induce further conformational changes and reorientations of melittin. Melittin, in a fully helical conformation, displays a bend of the two helical parts at the Pro residue.^18^ Due to the overlap of the IR signals (amide I’ band) from the two helical fragments, an average tilt of the helices in the membrane-bound melittin was calculated. At positive potentials, the long axis in the α-helices in melittin is perpendicular to the bilayer surface (the average tilt angle equals 0°); see Figure 7B,C. A negative potential shift leads to an increase in the average tilt angle of melittin helices to 10–15°. The potential-dependent change of the helix tilt is related to the emergence of the second amide I’ band at 1670 cm^–1^ (see Figures 6 and S7); the latter effect is related to the poorly hydrated carbonyl groups and disordered melittin fragments.

**Figure 8:**
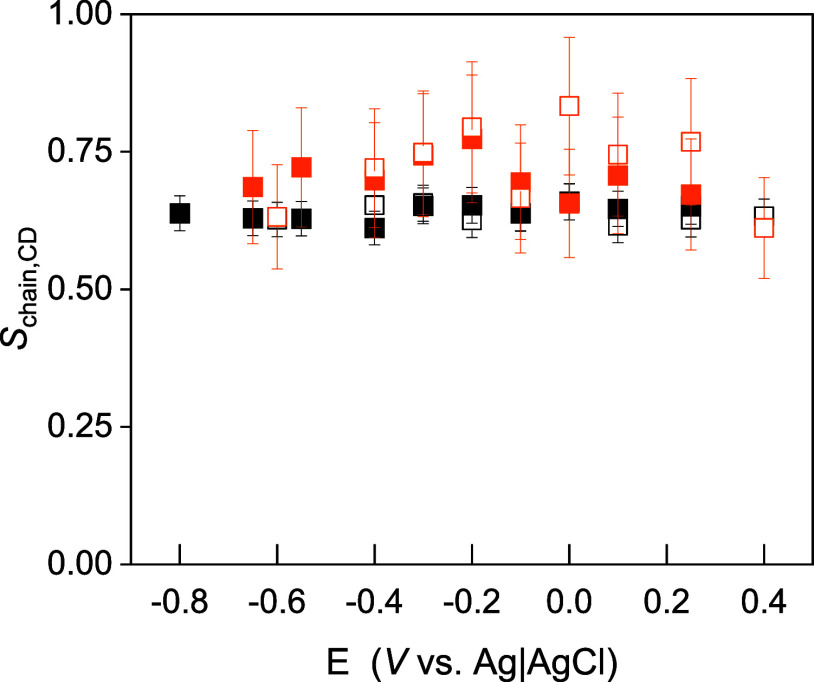
Order parameter (*S*_CD_) of the perdeuterated palmitoyl chain in the KLA- *d*_31_-POPE bilayer (black squares) and the order parameter for the bilayer exposed to 10 μM melittin for 15 min (orange squares). The electrolyte solution contained 50 mM KClO_4_ and 5 mM Mg(ClO_4_)_2_ in H_2_O. Full and empty symbols indicate the order parameter values determined from negative and positive potential scans, respectively.

To evaluate if melittin has an effect on the lipid molecules in both leaflets, *d*_31_-POPE lipid with a perdeuterated palmitoyl chain was used to fabricate the inner leaflet. The CD stretching modes appear in 2215–2070 cm^–1^ and are separated by ca. 700 cm^–1^ from the CH modes in the KLA and POPE lipids. Due to the conformational disorder of the acyl chains in *d*_31_-POPE and KLA lipids in the liquid-disordered OM, the exact tilt angle of the hydrocarbon chains cannot be determined.^64^ The quantitative analysis of some IR absorption modes may become complex due to the overlap of two factors: the conformational disorder of liquid chains and the molecular disorder caused by different orientations of an adsorbed molecule. The order parameter of the methylene groups depends on both types of disorder.^65^ The order parameter of the deuterated acyl chains (*S*_CD_) in the pure KLA- *d*_31_-POPE bilayer, as well as the *S*_CD_ for the bilayer exposed to 10 μM melittin for 15 min, were calculated and are shown in Figure 8.

The binding of melittin to the OM leads to a small increase in *S*_CD_, indicating that the peptide interacts with the hydrophobic fragment of *d*_31_-POPE in the inner leaflet. The increase in the *S*_CD_, as well as the previously reported increase in the *S*_CH_ in the KLA–POPE bilayer,^35^ indicates an ordering effect of melittin on the hydrophobic part of the OM. Thus, melittin insertion into the hydrophobic membrane region induces pores and/or channel formation in the bilayer that involves rearrangements of the KLA and POPE, leading to an improved packing of the lipids in the membrane.

Moreover, upon melittin binding to the OM, the average orientation of the acyl chains in KLA and POPE becomes independent of the electric potentials (membrane potentials).^35^ For membrane-associated melittin, the order parameter of the hydrocarbon chains in KLA and POPE lipids equals 0.65 ± 0.05, giving an approximate value of the tilt angle of the hydrocarbon chains between 26 and 32° versus surface normal. Due to the fact that the thickness of the hydrophobic part of the OM is constant during the potential scan, the lack of potential-dependent changes in the hydrophobic environment of the membrane is not responsible for the rearrangement of melittin at negative potentials. Furthermore, the impact of the external electric fields has a minimal impact on the melittin binding energy to the membrane surface. This can be estimated based on the characteristic values of the dipole moment of melittin and the external field used. Indeed, the dipole moment of melittin at the beginning of the three simulations is equal to 58 D. This value was calculated based on the structure and the partial charges of the peptide and is consistent with the one published earlier for solvated melittin.^66^ The characteristic external electric field value of 1 V yields an electric field value of 1.67 × 10^8^ V m^–1^ for the membrane thickness of 6 nm. These numbers yield a dipole contribution to the interaction energy of melittin with the external field on the order of 4.64 kcal mol^–1^, being negligible compared to the values shown in Figure 3, which is rooted in the Coulomb and dispersion interactions.

## Conclusions

MD simulations and PM IRRAS with electrochemical control reveal molecular-scale changes accompanying melittin interacting with a model OM. The interaction begins with a rapid formation of hydrogen bonds between the positively charged Lys21, Arg22, Lys23, and Arg24 residues at the C-terminus in melittin and hydroxyl, carboxylate, and phosphate residues in KLA in the outer leaflet of the OM. The formation of these hydrogen bonds immobilizes the α-helical melittin fragment in the polar region of the outer leaflet. In contrast, the N-terminus complex has a poorly defined structure and displays a large degree of flexibility. MD simulations reveal melittin penetration into the interfacial hydrophilic–hydrophobic region in the lipid A part of KLA. The simulations indicate an increase in the helical content in the melittin secondary structure upon the peptide binding to the membrane; this conclusion is in good agreement with the experimental PM IRRAS results. Over time, melittin interacting with the OM refolds and predominantly adopts the α-helical conformation. Such a conformational change occurs in melittin upon its insertion into the hydrophobic part of the OM.^3^ Adaptation of a helical conformation in melittin leads to its reorientation within the membrane; the helices align preferentially perpendicular to the membrane surface, making it possible for the flexible N terminal to anchor the peptide deeply into the acyl chains region of the OM. Spectroscopic evidence supporting this rationale follows from the isotopic substitution of POPE in the inner leaflet of the OM, where the average packing of the acyl chains is improved not only in KLA,^35^ but also in POPE. Thus, melittin has the ability to migrate from the polar region of the outer leaflet deep into the hydrophobic region of the inner leaflet in the OM.

Specific chemical groups in melittin and KLA directly involved in the peptide membrane association could be established through IRS. Interaction between melittin and the negatively charged carboxylate group in KLA was observed as a down-shift of the ν_as_(COO^–^) stretching mode in KLA, caused by a substitution of Mg^2+^ ions by an amide group of melittin. The removal of divalent ions from the saccharide fragment of lipopolysaccharides destabilizes the OM^34,36^ and aligns with the observed increase in the membrane capacitance. On the other hand, the reorientation of α-helices in membrane-bound melittin also contributes to an increase in the measured capacitance.

Despite an increase in the measured membrane capacitance, in the melittin-bound bilayer, the acyl chains have more compact packing, indicating that the peptide penetration into the OM does not dissolve the membrane. The effect could readily be explained by the exceptional conformational flexibility of melittin, as could be revealed by a tandem approach relying on spectroscopic measurements and atomistic MD simulations. The investigated melittin serves as a model case study for the AMP interaction with the cell envelope of Gram-negative bacteria. The mechanism deciphered here turns out to be vastly different and maybe even unique in comparison to the previously known AMP action on phospholipid bilayers.

## Methods

### Chemicals

1-Palmitoyl-2-oleoyl-*sn*-glycero-3-phosphoethanolamine (POPE), *d*_31_-1-palmitoyl-2-oleoyl-*sn*-glycero-3-phosphoethanolamine (*d*_31_-POPE), and di [3-deoxy-d-manno-octulosonyl]-lipid A (ammonium salt) Kdo_2_- lipid A (KLA) were purchased from Avanti Polar lipids. Lipids were used as received; no purification was done. Synthetic melittin (Cat. No.: M4171), Tris(hydroxy-d-methyl)-amino-*d*_2_-methan (*d*_5_-TRIS), KClO_4_ (99.99%), Mg(ClO_4_)_2_·6H_2_O (99%), and acid-sodium salt dihydrate (Na_2_EDTA) were purchased from Sigma-Aldrich (Germany); NaCl (99.5%) and MgCl_2_ (>99% p.a.) were purchased from Carl Roth (Germany); Tris(hydroxymethyl)aminomethane (TRIS) was purchased from Fluka (Germany); ethanol and methanol were purchased from AnalaR Normapur, VWR (France); and D_2_O was purchased from Euroisotop (Germany).

### Langmuir–Blodgett Transfer

Before each experiment, fresh lipid solutions were prepared. POPE was dissolved in CHCl_3_, while KLA was dissolved in CHCl_3_/CH_3_OH/H_2_O in a 13:6:1 volume ratio. The concentration of POPE equaled 1 μmol ml^–1^, and the concentration of KLA was 0.433 μmol ml^–1^ (1 mg mL^–1^). A microsyringe (Hamilton) was used to place several μLs of the lipid solution at the liquid|air interface of the Langmuir trough (KSV Ltd., Finland). All aqueous solutions were prepared from ultrapure water [resistivity 18.2 MΩ cm (PureLab Classic, Elga LabWater, Germany)]. Before compression, the lipid solution was left for 10 min for the solvent evaporation. Surface pressure vs area per molecule isotherms were recorded using the KSV LB mini trough (KSV Ltd., Finland) equipped with two hydrophilic barriers. Surface pressure was recorded as a function of the mean molecular area. The accuracy of these measurements was ±0.02 nm^2^ for the mean molecular area and ±0.1 mN m^–1^ for the surface pressure. Langmuir–Blodgett and Langmuir–Schaefer (LB–LS) transfers were used to prepare asymmetric supported planar KLA–POPE bilayers containing POPE in the inner (Au electrode oriented) and KLA in the outer (solution-oriented) leaflet on the gold surface; see Figure S1. Prior to the LB–LS transfer, each monolayer was compressed to the surface pressure of 30 mN m^–1^. First, the POPE monolayer was transferred from the aqueous subphase by vertical LB withdrawing at a rate of 15 mm min^–1^. The transfer ratio was 1.10 ± 0.10. The POPE monolayer transferred onto the Au substrate was left for 3 h. Next, a monolayer of KLA on 0.1 M KClO_4_ and 5 mM Mg(ClO_4_)_2_ aqueous subphase was compressed to the surface pressure of 30 mN m^–1^, and a horizontal LS transfer was used to fabricate the second leaflet on the gold surface, see Figure S1. Planar lipid bilayers were dried for at least 2 h before use in electrochemical and spectroelectrochemical experiments.

### Interaction of Melittin with Lipid Bilayers

The concentration of melittin in a buffer solution [20 mM Tris, 150 mM NaCl, and 5 mM EDTA (pH = 7.3 ± 0.1)] was set to equal either 1 or 10 μM. In such prepared solutions, melittin exists in the monomeric form.^67^ A gold electrode modified with a lipid bilayer was incubated in a hanging meniscus configuration in the buffer solution containing the monomeric form of melittin. For the melittin concentration of 1 μM, the incubation times were set to 15 min and 1 h. The incubation time from the 10 μM melittin solution was set to 15 min. After this time, the modified electrodes were carefully rinsed with water and used in either electrochemical or PM IRRAS experiments.

### Electrochemistry

Electrochemical measurements were performed in a glass three-electrode cell using a disc Au(111) single crystal (diameter 3 mm, MaTecK, Germany) as the working electrode in a hanging meniscus configuration. The surface roughness of the Au electrode was below 0.01 μm per 1 cm^2^ of the electrode surface. A gold wire was used as a counter electrode, and Ag|AgCl|sat.KCl (Ag|AgCl) was used as a reference electrode. All potentials are referenced against the Ag|AgCl|sat.KCl electrode. The electrolyte solution was 100 mM KClO_4_ with 5 mM Mg(ClO_4_)_2_. A Metrohm Autolab potentiostat (Metrohm Autolab, Holland) was used to perform the electrochemical measurements. Prior to the experiment, the cell was purged with argon for 1 h. The cleanliness of the electrochemical cell was tested by recording the cyclic voltammograms in the electrolyte solution. Alternative current voltammetry (ACV) was used to measure the capacitance of unmodified and KLA–POPE bilayer-modified Au(111) electrodes. AC voltammograms were recorded in negative and positive-going potential scans at a rate of 5 mV s^–1^ and the perturbation of the AC signal of 20 Hz and 10 mV amplitude. The differential capacitance versus potential curves were calculated from the in-phase and out-of-phase components of the AC signal, assuming that the cell was equivalent to a resistor in series with a capacitor.

### Polarization Modulation Infrared Reflection Absorption Spectroscopy

PM IRRA spectra were recorded using a Vertex 70 spectrometer with a photoelastic modulator (*f* = 50 kHz; PMA 50, Bruker, Germany) and a demodulator (Hinds Instruments). A homemade thin electrolyte layer spectroelectrochemical glass cell was washed in water and ethanol and placed in an oven (at 60 °C) for drying. CaF_2_ prism (optical window) was rinsed with water and ethanol and placed in a UV ozone chamber (Bioforce Nanosciences) for 10 min. The spectroelectrochemical cell has a built-in platinum counter electrode. The reference electrode was Ag|AgCl in 3 M KCl in either D_2_O or H_2_O. A disc Au(111) single crystal (diameter 15 mm, MaTecK, Germany) was used as the working electrode and mirror for the IR radiation. The surface roughness of the Au electrode was below 0.01 μm per 1 cm^2^ of the electrode surface. A lipid bilayer was transferred on the working electrode surface using LB–LS transfer. The electrolyte solution was 50 mM KClO_4_ with 5 mM Mg(ClO_4_)_2_ in D_2_O. The electrolyte solution was purged for 1 h with argon to remove oxygen. At each potential applied to the Au electrode, 400 spectra with a resolution of 4 cm^–1^ were measured. Five negative and five positive-going potential scans were recorded in each experiment. The negative going potential scan had the following potentials applied to the Au(111) electrode: 0.40, 0.25, 0.10, 0.00, −0.10, −0.20, −0.30, −0.40, −0.55, −0.65, and −0.80 V, while in the positive-going potential scan: −0.80, −0.60, −0.40, −0.30, −0.20, −0.10, 0.00, 0.10, 0.25, and 0.40 V. At each potential, the average, over five potential scans, spectrum was calculated and background corrected. The thickness of the electrolyte layer between the Au(111) electrode and the prism varied between 3 and 5 μm in different experiments. In one set of experiments, the half-wave retardation was set to 1600 cm^–1^ for the analysis of the amide I’ mode in melittin and C=O stretching mode in lipids. The angle of incidence of the incoming IR radiation was set to 55°. The solvent was D_2_O. For the analysis of the CD stretching modes in *d*_31_-POPE of the model OM, the half-wave retardation was set to 2100 cm^–1^. The angle of incidence was 52°. The solvent was H_2_O. All of the spectra were processed using OPUS v5.5 software (Bruker, Germany).

### Attenuated Total Reflection Infrared Spectroscopy

The analyte solution contained small unilamellar vesicles of the studied lipid mixture (KLA/POPE) (1.0:3.2 mol ratio) and 4.4 × 10^–4^ mol L^–1^ melittin. Three hundred microliters of 3.33 mg mL^–1^ POPE in chloroform and 300 μL of 3.33 mg mL^–1^ KLA in chloroform:methanol (2:1 vol) solutions were mixed and dried in a flow of argon. To remove the remaining solvent, the vials were placed in a vacuum desiccator for 72 h. Next, 500 μL of 20 mM *d*_5_-TRIS, 150 mM NaCl, and 5 mM MgCl_2_ in D_2_O was added to the dry lipid, and the mixture was sonicated (EMAG-Technologies, Germany) at 35 °C for 1 h. Afterward, 300 μL of synthetic melittin (Cat. No.: M4171) in 20 mM *d*_5_-TRIS, 150 mM NaCl, and 5 mM MgCl_2_ in D_2_O was mixed with the vesicle solution and left for interaction for 60 min at 35 °C giving the analyte solution. Attenuated total reflection infrared spectra were recorded by coadding 128 scans with a resolution of 4 cm^–1^ on a diamond prism using an MVP-Pro ATR unit (Harrick Scientific Products, Inc.) and the Bruker Vertex 70 spectrometer. The background spectrum was recorded for a drop (20 μL) of the electrolyte solution without lipid vesicles and melittin placed on the prism. The analyte spectrum was measured for a drop (20 μL) of the analyte solution (electrolyte with vesicles and melittin) placed on the prism. Subtraction of the analyte from the background spectrum gave the spectrum of lipids and melittin.

### Software Used for MD Simulations

MD simulations were carried out using NAMD 2.13^68,69^ with the CHARMM36 force field,^70−72^ utilizing explicit water solvent in a TIP3P model.^73^ The computational platform VIKING^74^ was used to set up the computations. The membrane system was designed using the CHARMM-GUIs^75−77^ bilayer membrane builder.^76,78−80^ Further preparation of the simulations and extraction of quantitative results, as well as the secondary structure analysis with STRIDE,^81^ were done using VMD.^82^

### MD Simulations of Solvated Melittin

The structure of the melittin monomer in its crystalline state was obtained from the melittin dimer available in the protein databank^83,84^ (PDB), with the ID 2mlt entry.^85^ In the crystalline state, melittin forms two α helices, which are separated by a bend occurring at the 14th residue; see Figure 1B. For the equilibration simulation, the melittin monomer was solvated in a cubical water box with a side length of 9 nm. The salt concentration used in the simulation resembled the experimental values of 50 mM KCl and 5 mM MgCl_2_, resulting in a total of 69,585 atoms in the system. The system was simulated for 100 ns, imposing periodic boundary conditions and a constant temperature of 303.15 K controlled by a Langevin thermostat.^68^ The simulation was split into 3 simulation stages. In the first stage, a 10 ns simulation was performed, employing the isothermal–isobaric statistical ensemble (NPT), maintaining a pressure of 1 bar using a Nosé–Hoover–Langevin piston pressure control^68,86^ with a time step of 1 fs for the integration of the equations of motion. The second and the third stages used canonical ensemble (NVT) with an integration time step of 1 and 2 fs and included simulations of 5 and 85 ns duration, respectively. No constraints were imposed on the atoms of the system during the three simulation stages (equilibration simulations). Explicit nonbonded interactions were neglected at 12 Å with a set smooth switching distance starting at 10 Å. Electrostatic interactions beyond 12 Å were calculated using the particle-mesh Ewald method.^87^ After the equilibration simulations, the system was further simulated for another 1 μs for data acquisition (production simulation). The acquired data was used as an independent control simulation of melittin in water.

### Equilibration of the Bilayer Membrane

The bilayer membrane was designed using the CHARMM-GUIs bilayer membrane builder.^75−77^ The chemical structure of the simulated OM matches the membrane studied in the experiments and is shown in Figure 1C. The negative charge at phosphate groups in the lipid A part in KLA was neutralized using Mg^2+^ ions, while the inner core in KLA using Na^+^ ions. The membrane had a density of 96.37 atoms nm^–3^. The number of lipids in the model OM is 34 KLA in the outer leaflet and 109 POPE lipids in the inner leaflet. Before the equilibration procedure, KLA and POPE molecules occupied an area of 1.95 nm^2^ and 0.61 nm^2^, respectively (see Section S2 and Figure S2); the converged equilibrated values appear 1.87 nm^2^ and 0.58 nm^2^, respectively. These values agree well with the LB–LS transfer conditions at which the average area per lipid *A*_KLA_ was 1.96 nm^2^ and *A*_POPE_ 0.67 nm^2^.^49^ The membrane was solvated in a water box, matching the membrane area of (8 × 8) nm^2^ and having a height of 11 nm. The concentration of KCl was assumed to be 50 mM. Due to the low amount of solvent the 5 mM MgCl_2_ used in the experiments, the Mg^2+^ ions were omitted in the simulation. NAMD^68,69^ was used to equilibrate the membrane for a total of 200 ns using an isothermal–isobaric ensemble and periodic boundary conditions in 3 distinct stages. The CHARMM36 force field^70−72^ was employed in the simulations. A constant temperature of 303.15 K controlled by a Langevin thermostat^68^ and a constant pressure of 1 bar using a Nosé–Hoover–Langevin piston pressure control^68,86^ was imposed during the whole simulation. The first stage was simulated for 5.375 ns with an integration time step of 1 fs. The second and the third stages used an integration time step of 2 fs each and were simulated for 2.125 and 90 ns, respectively. The simulations in stage 1 and stage 2 considered the movement of the head groups of all POPE and KLA lipids constrained using a harmonic restraint potential. The force constant of that restraint was decreased from 5 to 0.2 kcal mol^–1^ during the simulation stages 1 and 2. Explicit nonbonded interactions with a smooth switching starting at 10 Å were cut off at 12 Å, and electrostatic interactions were calculated using the particle-mesh Ewald method^87^ beyond the distance of 12 Å.

### Equilibrium Molecular Dynamics of Melittin Atop a Bilayer Membrane

**Figure 9:**
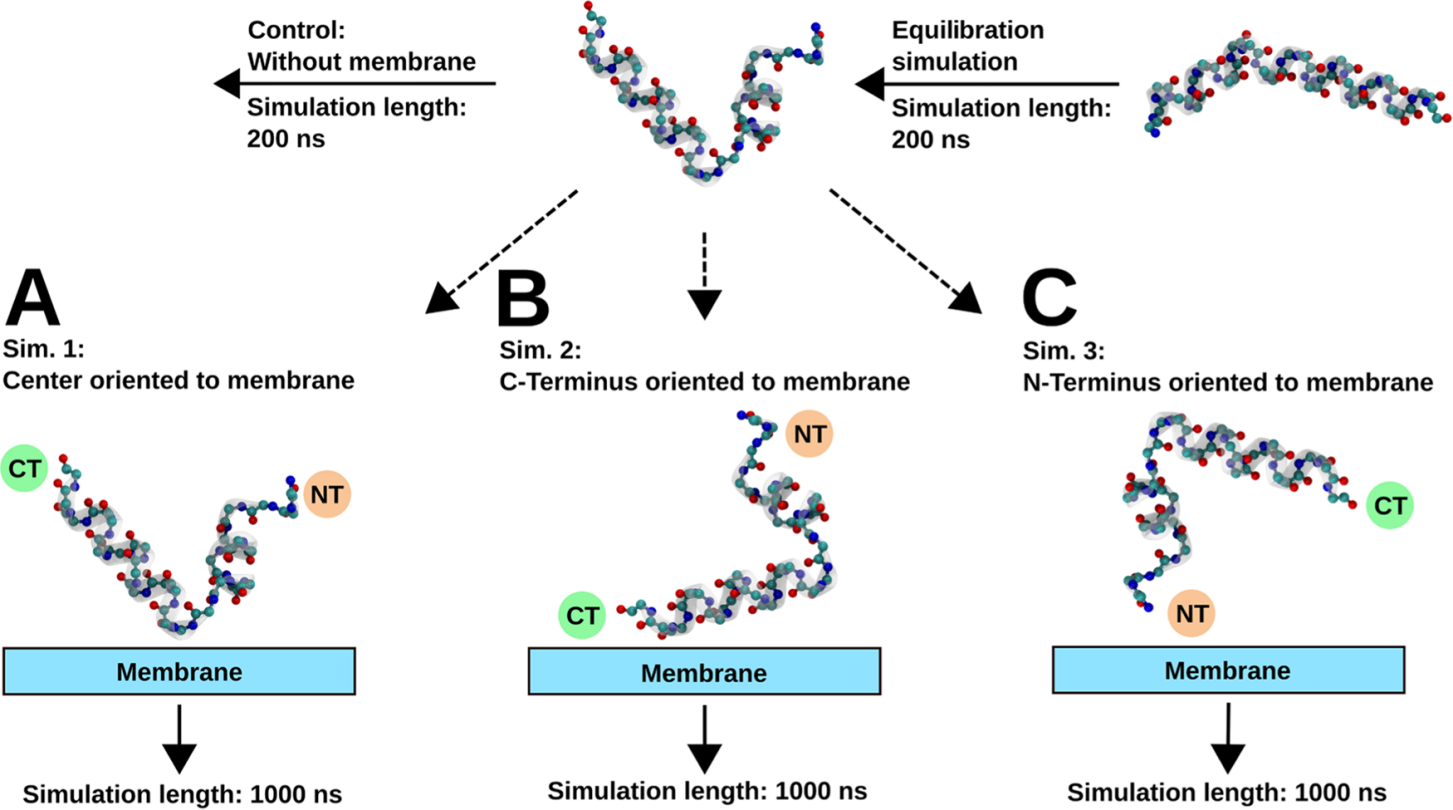
Schematic representation of the simulation procedure. The melittin peptide was equilibrated for 200 ns and solvated in a cubic water box. The resulting peptide conformation was used for four consecutive simulations. The peptide was placed on top of the equilibrated membrane in three different orientations facing the membrane’s surface: (A) with the bend in the peptide’s middle part, Sim. 1; (B) with its C-terminus, Sim. 2; and (C) N-terminus, Sim. 3. An independent control simulation of melittin without a membrane solvated in a cubic water box was also carried out (upper panel). Water is omitted from the graphical representations for clarity.

A combined system, shown in Figure 1A, of the equilibrated melittin monomer and the equilibrated membrane was used to generate the data for analysis. The melittin monomer, which acquired a V-shape during the equilibration simulation, was placed on top of the membrane in three different initial orientations, depicted in Figure 9. Simulation 1 (Sim. 1) considered melittin placed with its helix bend 1.2 nm above the membrane surface (Figure 9A). Simulation 2 (Sim. 2, Figure 9B) and simulation 3 (Sim. 3, Figure 9C) had melittin placed with the C-terminus and the N-terminus nearest to the membrane, respectively. The electrolyte solution contained 50 mM KCl. These composite systems with a total number of atoms varying between 82,399 and 82,405 were simulated with periodic boundary conditions. After the composite systems were constructed, each system was first equilibrated for 5 ns in the isothermal–isobaric ensemble, maintaining a pressure of 1 bar using a Nosé–Hoover–Langevin piston pressure control^68,86^ and a constant temperature of 303.15 K using a Langevin thermostat,^68^ followed by a 5 ns simulation in the canonical ensemble with an integration time step of 1 fs. After the equilibration simulation, production simulations of each system with a duration of 1 μs in the canonical ensemble with an integration time step of 2 fs were performed. A 12 Å cutoff was imposed for the nonbonded interactions, which were linearly switched to zero starting from 10 Å. Beyond these distances, electrostatic interactions were calculated using the particle-mesh Ewald method.^87^

## Supporting Information Available

Formation of the asymmetric models’ outer membrane of gram-negative bacteria; area per lipid of the outer membrane and volume of the simulation box during the equilibration simulation; possible orientation of melittin on the membrane surface and their effect on the capacitance of a model membrane deposited on an electrode surface; calculation of electrostatic and van der Waals contribution to the interaction energy; secondary structure analysis of melittin interacting with the outer membrane; location and conformation of melittin associated with the KLA–POPE bilayer; deconvolution of the amide I’ mode in melittin; PM IRRA spectra of the KLA–POPE bilayer after interaction with 1 μM melittin; and determination of the helix tilt angle from the PM IRRA spectra (PDF)
