# Supplementary material for: How do Antimicrobial Peptides Interact with the Outer Membrane of Gram-Negative Bacteria? Role of Lipopolysaccharides in Peptide Binding, Anchoring, and Penetration
Source: ACS Infect Dis. 2024 Jan 23;10(2):763–78. doi: 10.1021/acsinfecdis.3c00673 (PMC10862549; doi:10.1021/acsinfecdis.3c00673)
Supplement: Supplementary file 1 — id3c00673_si_001.pdf [file id3c00673_si_001.pdf]

Supporting Information

How do Antimicrobial Peptides Interact with the Outer Membrane of Gram-Negative Bacteria? Role of Lipopolysaccharides in the Peptide Binding, Anchoring and Penetration

Justus C. Stephani[1], Luca Gerhards[1], Bishoy Khairalla[2,5], Ilia A. Solov'yov[1,3,4]*, and Izabella Brand[2]*

[1] *Institute of Physics, Carl von Ossietzky University of Oldenburg, 26111 Oldenburg, Germany*

[2] *Department of Chemistry, Carl von Ossietzky University of Oldenburg, 26111 Oldenburg, Germany*

[3] *Research Center Neurosensory Science, Carl von Ossietzky University of Oldenburg, 26111 Oldenburg, Germany*

[4] *CeNaD – Center for Nanoscale Dynamics, Carl von Ossietzky University of Oldenburg, 26111 Oldenburg, Germany*

[5] *Present address: Friedrich-Alexander-Universität Erlangen-Nürnberg, Department of Biology, Pharmaceutical Biology, Staudtstr. 5, 91058 Erlangen, Germany*

**Corresponding authors:* izabella.brand@uni-oldenburg.de; ilia.solovyov@uni-oldenburg.de

S1. Formation of the asymmetric model outer membrane of Gram-negative bacteria

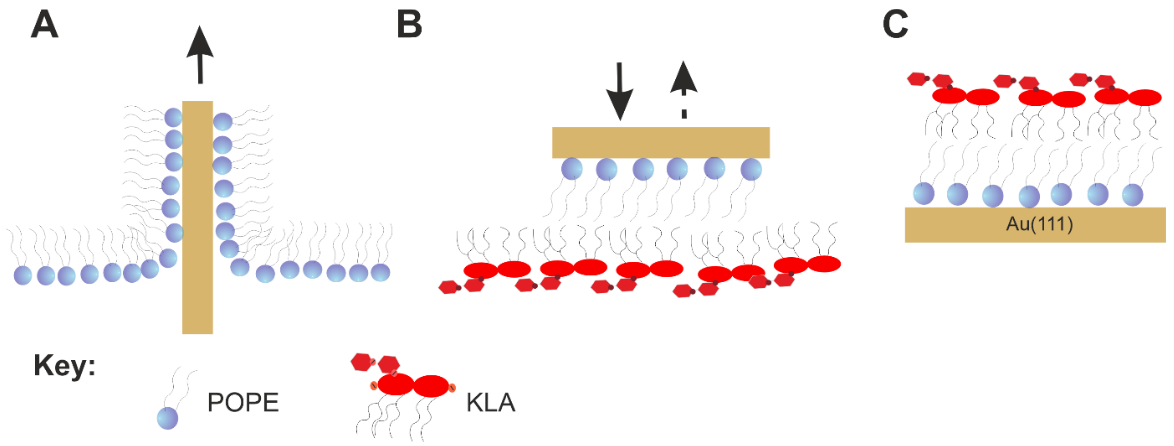

Figure S1. Illustration of the fabrication procedure of the asymmetric KLA-POPE model outer membrane of Gram-negative bacteria **A**: transfer of the inner POPE leaflet by Langmuir-Blodgett vertical withdrawal, **B**: transfer of the outer KLA leaflet by Langmuir-Schaefer method and **C**: molecular scale order in the LB-LS transferred bilayer.

Langmuir-Blodgett (LB) and Langmuir-Schaefer (LS) transfers were used to prepare asymmetric KLA-POPE bilayers on a gold surface. First, a POPE (or d_{31}-POPE) monolayer was transferred from the aqueous subphase by a vertical LB withdrawal, see Fig. S1A. Withdrawal of a hydrophilic gold substrate from the aqueous subphase through the air|water interface covered by the phospholipid monolayer gave the inner leaflet of the model outer membrane. Next, a monolayer of KLA on a 0.1 M $KClO_4$ and 5 mM $Mg(ClO_4)_2$ aqueous subphase was compressed to the surface pressure $\Pi = 30$ mN m^{-1} and a horizontal LS transfer was used to fabricate the second leaflet of the model outer membrane, see Fig. S1B. During this transfer, the hydrophobic surface of the POPE modified gold surface and was exposed to the hydrophobic hydrocarbon chains in the KLA monolayer present at the aqueous electrolyte|air interface. The gold substrate was covered by a Y-type lipid bilayer as shown schematically in Fig. S1C.

S2. Area per lipid of the outer membrane and volume of the simulation box during the equilibration simulation

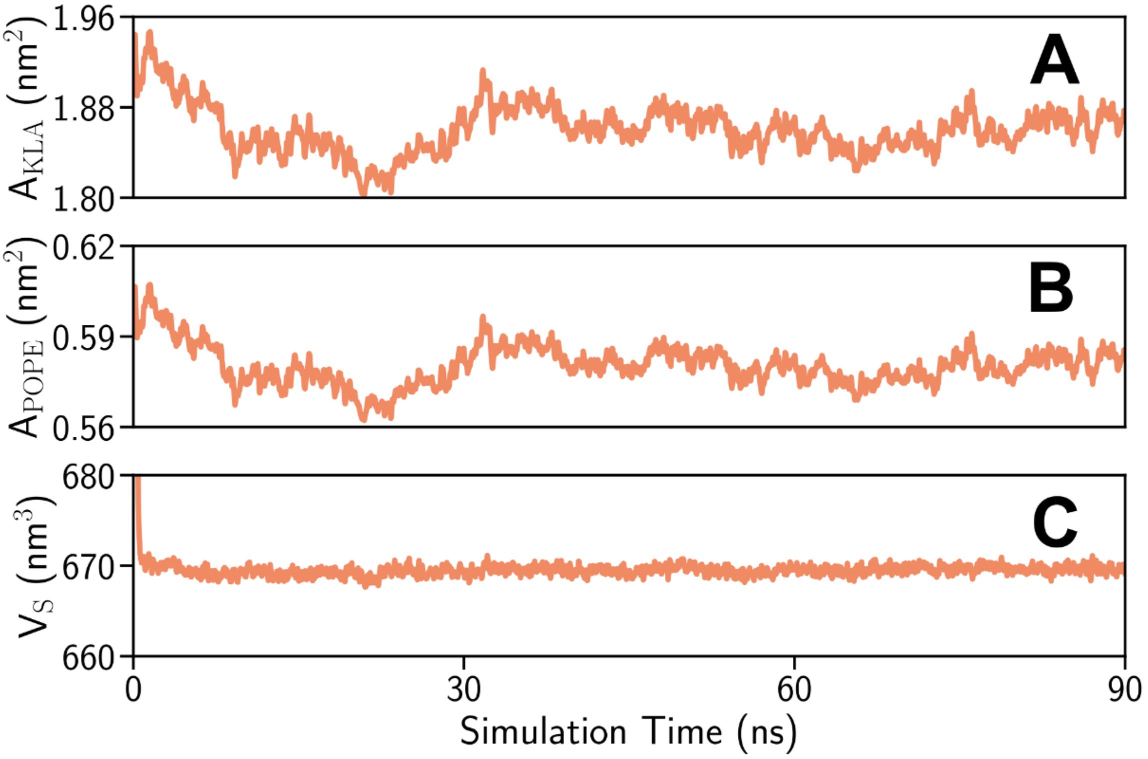

Figure S2. **A**: Time evolution of the average area per KLA lipid in the outer leaflet of the model outer membrane; **B**: Time evolution of the average area per POPE phospholipid on the inner leaflet of the model outer membrane. The values were computed as an average over all lipids present in the simulation box. **C**: Volume of the simulation box V_S over simulation time.

During the equilibration process of the model outer membrane, the volume of the simulation box and the average area per lipid attributed to KLA and POPE molecules decreased. Figure S2A shows the average area per KLA lipid in the outer leaflet of the membrane over simulation time, while Fig. S2B shows the average area per POPE phospholipid in the inner leaflet of the outer membrane. The average areas per lipid A_{KLA} and A_{POPE} converge to the values 1.87 nm^2 and 0.58 nm^2, respectively. These values agree well with the LB-LS transfer conditions at with the average area per A_{KLA} was 1.96 nm^2 and A_{POPE} – 0.67 nm^2.[1] The time evolution of the total volume of the simulation box V_S is shown in Fig. S2C.

S3. Possible arrangements of melittin on the membrane surface and their effect on the capacitance of a model membrane deposited on an electrode surface

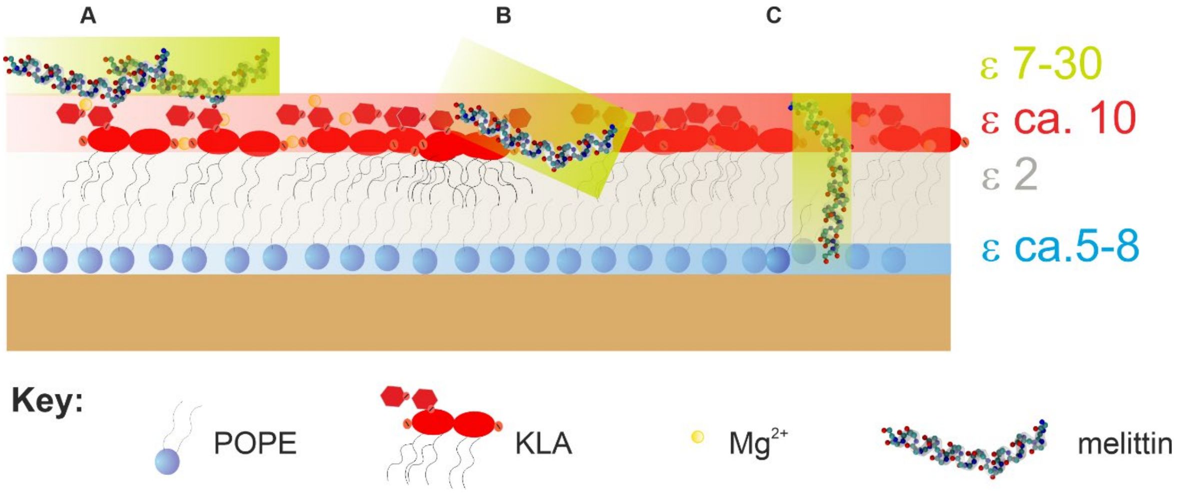

Figure S3. Schematic structure of the KLA-POPE model outer membrane on a gold electrode surface with possible arrangements of melittin interacting with the membrane from solution **A**: parallel **B**: tilted and **C**: perpendicular orientations. The dielectric constants of polar head groups and hydrocarbon chains in lipids as well as of the peptide are given in the figure.

Figure S3 shows possible arrangements of melittin in the model outer membrane of Gram-negative bacteria.

The capacitance of a film modifying an electrode surface is

$$C = \frac{\varepsilon_0 \varepsilon A}{d} , \qquad \text{(S1)}$$

where ε_0 is the permeability of vacuum, ε is the dielectric constant of the film at the electrode surface, d is the thickness of the film, and A is the surface area of the electrode. The only purpose of presenting this equation is due to the description of the dependence of the measured capacitance on the dielectric constants of molecules present in the film.

Our previous studies [2] demonstrated that the tilt of the acyl chains in the KLA-POPE bilayer with bound melittin do not change during the potential scan indicating that the OM thickness remains constant. Therefore, changes in the OM do not contribute to the measured capacitance. Adsorption of a protein on top of the OM, could potentially lead to a decrease in the

capacitance, however the effect of the dielectric constant is stronger than of the membrane thickness changes.

S4. IR spectra of KLA : melittin-POPE bilayer

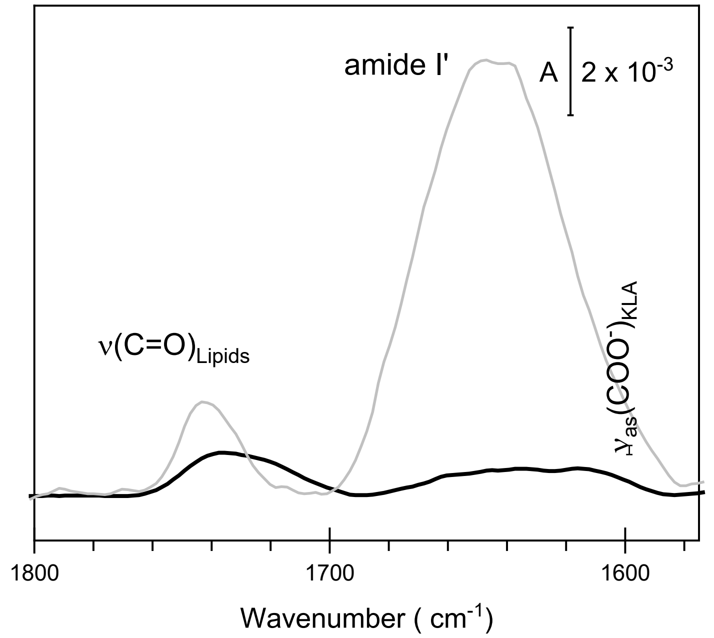

Figure S4. PM IRRA spectra of the KLA:Mel (9:1 mole ratio)-POPE bilayer directly after the LB-LS transfer onto the Au(111) surface, ex situ spectrum measured at the air|Au interface (gray line) and after immersion of the monolayer into the electrolyte solution, in situ measured spectrum at the D_2O|Au interface (black line). In situ measurements were carried out in 50 mM $KClO_4$ and 5 mM $Mg(ClO_4)_2$ in D_2O. The absorbance is shown in arbitrary units.

Figure S4 shows PM IRRA spectra of a freshly transferred KLA : Mel (9:1 mole ratio)-POPE bilayer and after its immersion into the electrolyte solution. The PM IRRA spectra in Fig. S4 feature the ν(C=O) stretching modes in the ester carbonyl groups in lipids, amide I' vibration mode mainly in melittin and $\nu_{as}(COO^{-})$ stretching mode in KLA. The PM IRRA spectrum of a freshly LB-LS transferred KLA:Mel (9:1 mole ratio)-POPE bilayer gives a strong signal in 1700 – 1600 cm^{-1}, which arises from the amide I' vibrational mode in melittin, see gray line Fig. S4. Immersion of the bilayer into the electrolyte solution cased a dramatic decrease in the intensity of the amide I' mode, see black line in Fig.S4. This result indicates a dissolution

of a significant fraction of melittin from the membrane surface into the electrolyte phase. Note, that the ν(C=O) absorption band in lipids underwent a down-shift, due to a better hydration of the ester carbonyl groups in the KLA and POPE molecules upon immersion in D_2O.[3, 4] However, the integral intensity of the ν(C=O) absorption band did not change, indicating no loss of the lipid molecules upon immersion of the bilayer into the electrolyte solution.

S5. Calculation of electrostatic and disperse (van der Waals) contributions to the interaction energy

The electrostatic contribution to the interaction energy of a residue *x* in the peptide with the membrane is calculated as:

$$U_{\mathrm{elec}}(x) = \frac{1}{4\pi\varepsilon_0} \Sigma_{(i=1)}^{N} \Sigma_{(j=1)}^{M} \frac{q_i q_j}{r_{ij}} \ , \tag{S2}$$

where r_{ij} is the distance between two charges q_i and q_j, where the first summation goes over all the *N* atoms of the residue of interest, while the second summation goes over all the *M* atoms of the membrane. The dispersive (van der Waals) contribution to the interaction energy could be calculated using the Lennard-Jones potential as

$$U_{vdW}(x) = \sum_{i=1}^{N} \sum_{j=1}^{M} \varepsilon_{ij} \left[\left(\frac{\sigma_{ij}}{r_{ij}} \right)^{12} - 2 \left(\frac{\sigma_{ij}}{r_{ij}} \right)^{6} \right], \tag{S3}$$

where r_{ij} is the distance between two atoms *i* and *j*, where the first summation goes over all the *N* atoms of the residue of interest, while the second summation goes over all the *M* atoms of the membrane. ε_{ij} and σ_{ij} are the equilibrium van der Waals energy and distance for a given pair of atoms.

S6. Secondary structure analysis of melittin interacting with the outer membrane

The secondary structure of the peptide was determined using the STRIDE algorithm in VMD.[5, 6] Helicity is defined as the amount of α-, 3_{10}- and π-helices in the secondary structure of the peptide. The time dependency of the helicity is plotted in Fig. S5A for the three production simulations of the composite systems (Sim. 1 - Sim. 3) and the control simulation of melittin in water (Control). The helicity value decreased most notably in the Control simulation from 75% to 35% during the simulation time of 1000 ns. In Sim. 1 the helicity decreased slightly from 75% to 70% during the whole simulation, whereas in Sim. 2 the unfolding of the helical

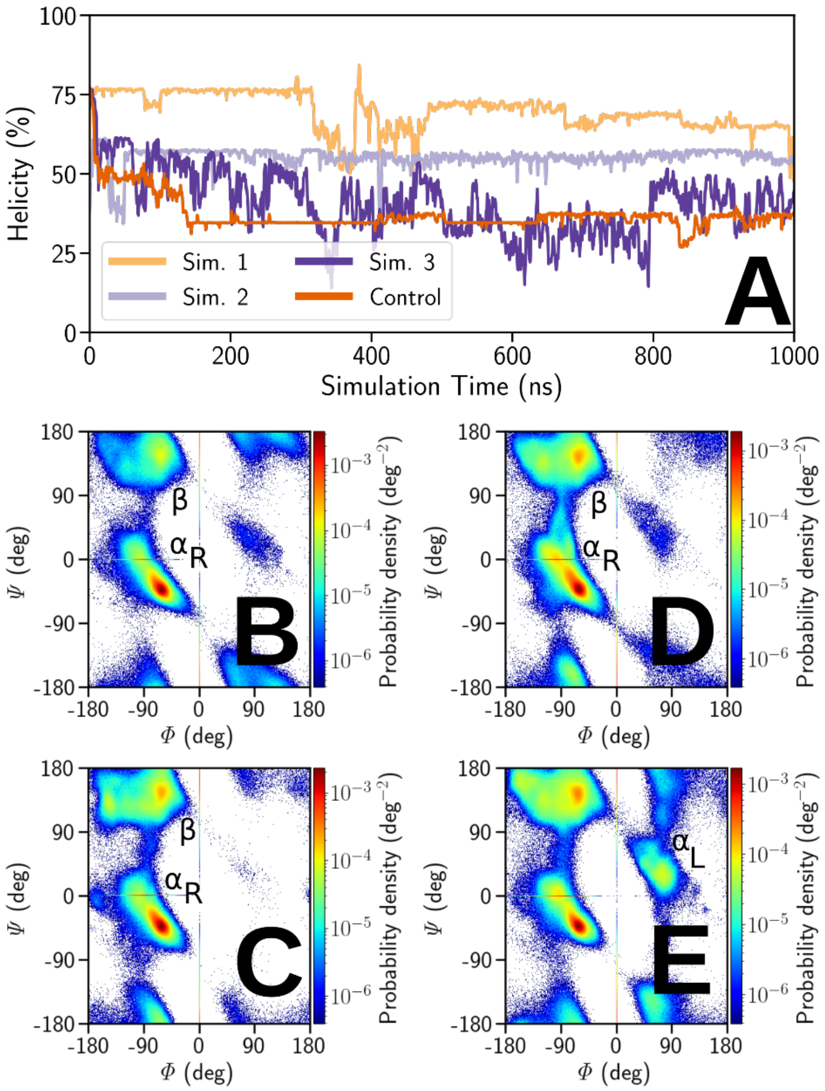

Figure S5. A: Time dependency of helicity of melittin (percent) for the four simulations Sim. 1, Sim. 2, Sim. 3 and Control. **B-E**: Probability distributions of the backbone dihedral angles ψ and φ computed for Sim. 1 (**B**), Sim. 2 (**C**), Sim. 3 (**D**) and Control (**E**). The white area corresponds to the sterically forbidden conformations of the peptide**.**

structures is concentrated in the first 100 ns of the simulation time and the helicity remained largely constant at 60% for the rest of the simulation time. The results indicate that the interaction of the peptide with the membrane stabilizes the helical structures. On the other hand, the rapid changes in helicity throughout the simulation time, as shown in the results of Sim. 3 indicate a high conformational flexibility of the peptide.

Figure S5B-C show the Ramachandran plots of the probability distribution of the backbone dihedral angles ψ and φ computed for Sim. 1, Sim. 2, Sim. 3 and Control, respectively. While in Sim. 1-3 the right turning α-helix is the dominant secondary structure followed by the β-

sheet, in the Control simulation the probability to observe α-helix decreases and other conformations are possible, further indicating the unfolding of the α-helix of melittin in solution.

S7. Location and conformation of melittin associated with the KLA-POPE bilayer

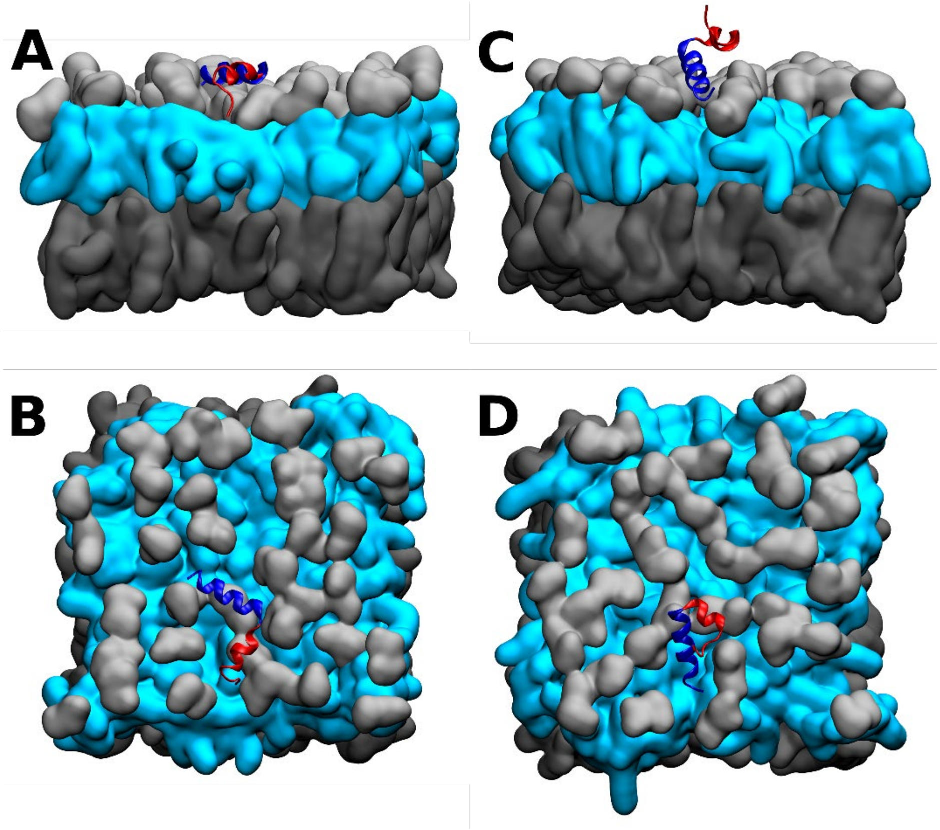

Figure S6. Characteristic configurations of melittin bound to the model outer membrane. The N- and C-terminus are shown in red and blue, respectively. The lower leaflet of the outer membrane is shown in gray, while the lipid A part of KLA in the upper leaflet is shown in cyan and the inner core region of KLA is shown in silver. Each panel shows a snapshot of melittin atop the membrane, once from the side and once from the top. The snapshots are taken from Sim. 1 after approximately 30 ns (**A/B**) and 600 ns (**C/D**).

Figure S6 illustrates characteristic arrangements of melittin in atop the membrane. Panels A and B display the melittin state after a 30 ns simulation time, where the N-terminus, depicted in red, has shifted beyond the inner core of KLA and adheres to the lipid A. However, it has not infiltrated the amphiphilic region of lipid A. Panels C and D depict the melittin state after

a 600 ns of simulation time. At this stage, the N-terminus has detached while the C-terminus remains bonded to the membrane.

S8. Deconvolution of the amide I' band in melittin

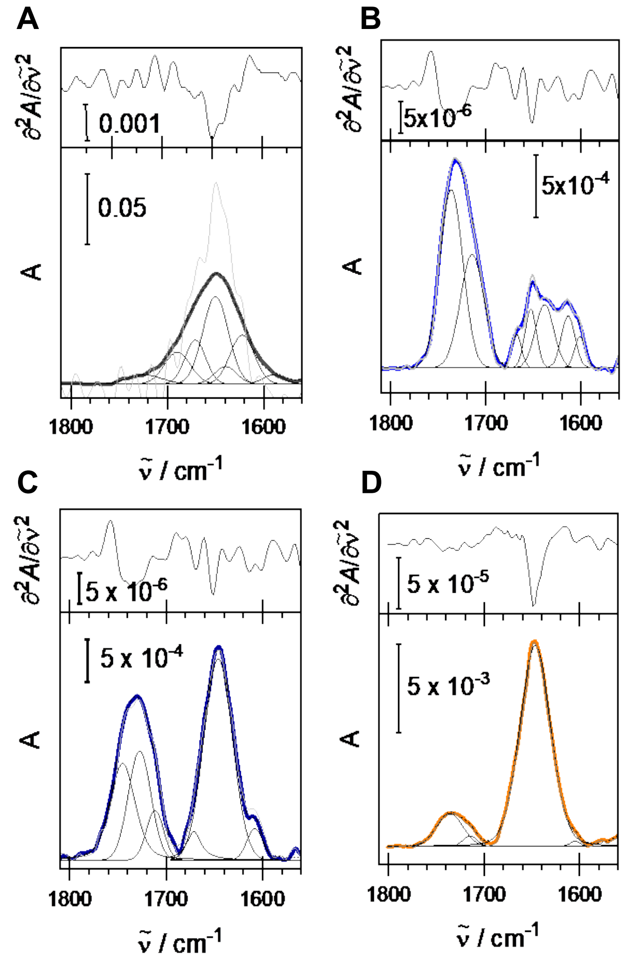

Figure S7. Upper panel: the second derivative of the measured IR spectra and the bottom panel: corresponding IR spectra (thick color lines) KLA-POPE lipid systems after interaction with melittin. Thin black lines show the band deconvolution results. **A**: attenuated total reflection IR spectrum of 4.4 × 10^{-4} M melittin after 1h of interaction with KLA-POPE vesicles, **C-D**: PM IRRA IR spectra of the KLA-POPE bilayer at *E* = 0.0 V vs Ag|AgCl after **B**: 15 min interaction with1 µM melittin, **C**: 1h interaction with 1µM melittin and **D**: 15 min interaction with 10 µM melittin. All spectra were recorded in 50 mM $KClO_4$ and 5 mM $Mg(ClO_4)_2$ in D_2O. The scale bars show absorbance in arbitrary units.

The second derivative and Fourier self-deconvolution were used to deconvolute the measured IR spectra. Fourier self-deconvolution was done to fit the measured amide I' band with Gauss

shaped curves with the full width at half maximum set to 23 cm^{-1}. The wavenumbers of the second derivative minima overlapped with the position of the maxima of the Fourier self-deconvoluted bands yielding the number of the deconvoluted bands. The measured IR spectra were deconvoluted using OPUS software (Bruker, Germany) by setting the number of amide I' band components and their positions (wavenumbers) to the results of the second derivative and Fourier self-deconvolution. The individual components of the amide I' band were fitted with Gauss curves. Figure S7 shows the deconvoluted measured IR spectra (bottom panels), the Fourier self-deconvoluted spectra (bottom panels, gray lines), and the second derivatives of the experimental spectra (top panel) of the KLA-POPE lipids upon interaction with melittin.

S9. PM IRRA spectra of the KLA-POPE bilayer after interaction with 1 µM melittin

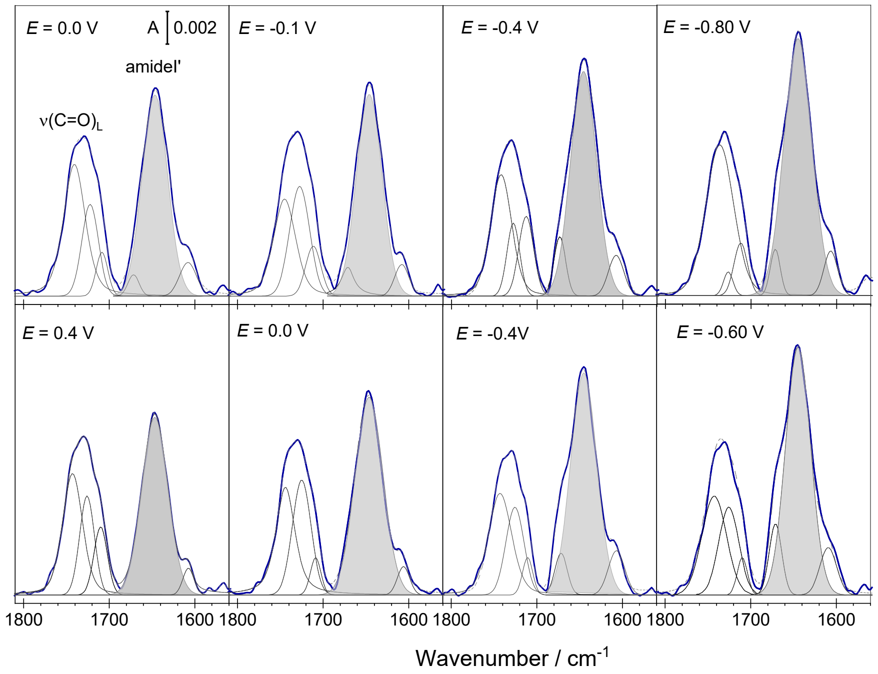

Figure S8. IR spectra of KLA-POPE bilayers on Au(111) after 1 h of interaction with 1 µM melittin (dark blue lines) recorded at different electrode potentials. The upper and lower panels show results for the negative and positive scans, respectively. Thin black lines show the band deconvolution results. The bands highlighted in gray show the amide I' mode of α-helices in melittin. Measurements were carried out in 50 mM $KClO_4$ and 5 mM $Mg(ClO_4)_2$ in D_2O. The absorbance is shown in arbitrary units.

Figure S8 shows the PM IRRA spectra of the KLA-POPE bilayer exposed to 1 µM melittin solution for 60 minutes.

S10. Determination of the helix tilt angle from the PM IRRA spectrum

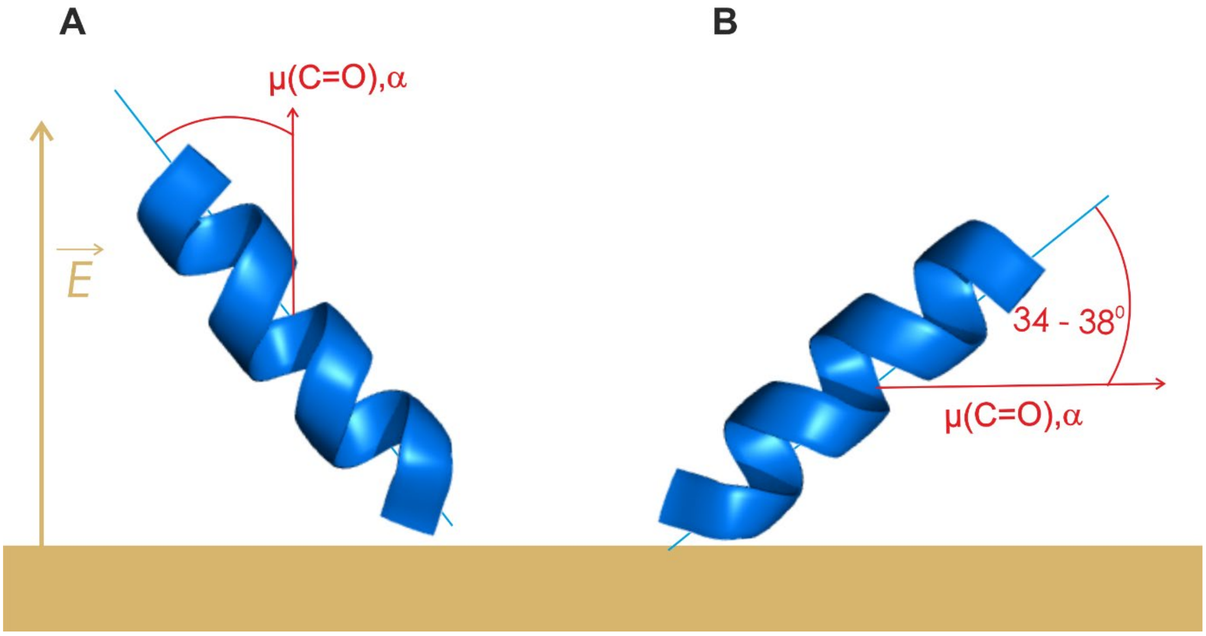

Figure S9. Limiting cases for the orientation of an α-helical peptide fragment adsorbed on a solid surface in which the transition dipole moment of the amide I' band is **A**: parallel and **B**: normal to the direction of the electric field vector of the reflected IR radiation. The blue lines show the direction of the long axis of the α-helix. The direction of the transition dipole vector of the amide I' mode (red arrow) and the direction of the electric field vector of the *p*-polarized light at the phase boundary (gold arrow) are shown in the figure.

According to the surface selection rule of IRRAS[7, 8], in an anisotropic film, the intensity of an IR absorption mode depends on the surface concentration of species adsorbed on a solid surface and on the average orientation of a given transition dipole vector $\left(\vec{\mu}\right)$ vs. electric field $\left(\vec{E}\right)$ (surface normal), characterized by the angle θ. In a PM IRRA experiment an average orientation of a given functional group can be calculated form the $\langle\theta\rangle$ value. Depending on the orientation of the molecule in a film, some IR absorption bands are enhanced while others attenuated in the IRRA spectrum. Figure S9 shows two limiting cases for the orientation of an α-helical fragment in a peptide adsorbed on a solid surface leading to the enhancement and cancellation of the amide I' band intensity in the IRRA spectrum. A parallel orientation of the $\vec{\mu}$ and $\vec{E}$ vectors causes their strong coupling enhancing the intensity of the IR absorption band of the amide I' band (Fig. S9A).

Once the angle between the $\vec{\mu}$ and $\vec{E}$ vectors equals 90 º (Fig. S9B) the integral intensity of the amide I' band equals zero. In this case there is no coupling of the transition dipole and the electric field vectors.

For a peptide where the α-helical fraction is known, one can calculate the $\langle\theta\rangle$ between the transition dipole moment $\vec{\mu}$ of the deconvoluted amide I' band of α-helices relatively to the surface normal, being colinear with the direction of the electric field vector

$$\cos^2\langle\theta\rangle = \frac{1}{3}\frac{c_{Ex}}{c_{\mathrm{R}}}. \tag{S4}$$

Here c_{Ex} corresponds to the percent content of the amide I'α-helix band in the entire amide I' band in a measured PM IRRA spectrum and the c_{R} is the percent content of the α-helical structures in the solution phase (random distribution). The angle $\langle\theta\rangle$ can be used to calculate the order parameter S as follows[9]

$$S = \left\{\frac{1}{2}\left(3\left(\cos^2\langle\theta\rangle\right)-1\right)\right\}. \tag{S5}$$

Finally, the order parameter of the long axis of the α-helix (S_{helix}) can be calculated as

$$S_{helix} = \frac{2S}{3\cos^2\alpha - 1}, \tag{S6}$$

where α is the angle between the long axis of the α-helix and the transition dipole moment of the amide I' band of α-helices. In an α-helical protein fragment the transition dipole vector of the amide I' mode $\vec{\mu}$ makes an angle of 34 ° – 38 ° vs. the long axis of the α-helix[10, 11], see Fig. S9. The order parameter S_{helix} was used to calculate the tilt of the helix with respect to the surface normal ($\mathrm{Tilt}_{\mathrm{helix}}$).

References

(1) Khairalla, B.; Brand, I. Membrane potentials trigger molecular-scale rearrangements in the outer membrane of Gram-negative bacteria. *Langmuir* **2022**, *38*, 446−457.

(2) Brand, I.; Khairalla, B. Structural changes in the model of the outer cell membrane of Gram-negative bacteria interacting with melittin: an in situ spectroelectrochemical study. *Faraday Discuss.* **2021**, *232*, 68-85.

(3) Blume, A.; Hübner, W.; Messer, G. Fourier transform infrared spectroscopy of $^{13}C{=}O$ labeled phospholipids hydrogen bondong to carboxyl groups. *Biochemisty* **1988**, *27*, 8239-8249.

(4) Zawisza, I.; Wittstock, G.; Boukherroub, R.; Szunerits, S. PM IRRAS investigation of thin silica films deposited on gold. Part 1. Theory and proof of concept. *Langmuir* **2007**, *23*, 9303-9309.

(5) Frishman, D.; Argos, P. Knowledge-bsed protein secondary structure assignment. *Proteins: Struct. Funct. Bioinform.* **1995**, *23*, 566-579

(6) Humphrey, W.; Dalke, A.; Schulten, K. VMD: Visual Molecular Dynamics. *J. Mol. Graphics* **1996**, *14*, 33-38

(7) Moskovits, M. Surface selection rules. *J. Chem. Phys.* **1982**, *77*, 4408-4416.

(8) Brand, I. *Application of Polarization Modulation Infrared Reflection Absorption Spectroscopy in Electrochemistry*; Springer Nature, 2020.

(9) Seelig, A.; Seelig, J. The dynamic structure of fatty acyl chains in a phospholipid bilayer measured by deuterium magnetic resonance. *Biochemisty* **1974**, *13*, 4839-4845.

(10) Tsuboi, M. Infrared dichroism and molecular conformation of α-form poly-γ-benzym-L-glutamate. *J. Polym. Sci.* **1962**, *59*, 139-153.

(11) Miyazawa, T.; Blout, E. R. The infrared spectra of polypeptides in various conformations: amide I and II bands. *J. Am. Chem. Soc.* **1961**, *83*, 712-719.
